# Ivermectin inhibits epithelial-to-mesenchymal transition via Wnt signaling in endocrine-resistant breast cancer cells

**DOI:** 10.1371/journal.pone.0326742

**Published:** 2025-06-26

**Authors:** Kitiya Rujimongkon, Patthamapon Adchariyasakulchai, Phum Meeprasertskul, Wannarasmi Ketchart

**Affiliations:** Department of Pharmacology, Faculty of Medicine, Chulalongkorn University, Bangkok, Thailand; University of the Punjab Quaid-i-Azam Campus: University of the Punjab, PAKISTAN

## Abstract

Ivermectin (IVM), an antiparasitic drug, has been explored for its anticancer properties in various cancer types, including breast cancer. Endocrine therapy resistance poses a significant challenge in breast cancer treatment, often leading to metastasis prevention failure. This study aimed to investigate the effects of IVM on endocrine-resistant breast cancer cells, focusing on mechanisms associated with epithelial-to-mesenchymal transition (EMT). IVM was administered to endocrine-resistant breast cancer cell lines, MCF-7/LCC2 (tamoxifen resistant) and MCF-7/LCC9 (fulvestrant resistant), to evaluate its influence on cell proliferation, invasion, and EMT-related mechanisms. The findings indicated that IVM’s half-maximum inhibitory concentration (IC_50_) inhibited MCF-7/LCC2 and MCF-7/LCC9 at 9.35 and 9.06 µM, respectively, within 24 h of treatment. Moreover, IC_50_ concentration treatment for 24 h led to over a 50% reduction in cell motility and a 62% and 35% decrease in cell invasion in MCF-7/LCC2 and MCF-7/LCC9 cells, respectively. Metastasis biomarkers demonstrated that IVM treatment reduced the expression of vimentin and snail. The study also discovered that IVM diminished the expression of Wnt5a/b and lipoprotein receptor-related protein 6 (LRP6), associated with the metastasis-related Wnt signaling pathway. In conclusion, IVM inhibits the Wnt signaling pathway associated with EMT in the metastasis of endocrine-resistant breast cancer cells. These insights underscore the potential of repurposing IVM for endocrine-resistant breast cancer patients.

## Introduction

Ivermectin (IVM), a macrocyclic lactone group member, is clinically, used as an antiparasitic treatment for onchocerciasis and intestinal strongyloidiasis in humans for over four decades [1]. IVM’s activities also exhibit antiviral properties in flavivirus, HIV-1, dengue, and SARS-CoV-2 [2–4]. Beyond infectious diseases, IVM has demonstrated potency in various types of cancers such as breast cancer, gastric cancer, hepatocellular carcinoma, renal cell carcinoma, prostate cancer, leukemia, cervical cancer, ovarian cancer, glioma, lung cancer, nasopharyngeal cancer, and melanoma [5–10]. In breast cancer studies, IVM inhibited cell proliferation in ER-positive (MCF-7) and triple-negative breast cancer (TNBC) (MDA-MB-231) cells via the Akt/mTOR pathway, leading to autophagy [6]. IVM suppressed metastasis by modulating the transcription of epithelial-to-mesenchymal transition (EMT) genes in MDA-MB-231 cells [11]. IVM enhanced inflammation by inducing T-cell infiltration into breast tumors via the modulation of the P2X4/P2X7/Pannexin-1 pathway [12]. IVM arrested the cell cycle by modulating associated cell cycle control in breast cancers [13]. IVM suppressed breast cancer cell migration by inhibiting Wnt signaling pathway [14]. Thus, IVM exhibits various anticancer mechanisms in breast cancer cells.

The Wnt signaling pathway, crucial in breast cancer, includes two primary cascades: the canonical (β-catenin-dependent) and noncanonical (independent) pathways [15]. This pathway is linked to cancer progression, involving cell proliferation and the EMT process of metastasis [16]. Numerous studies have shown the correlation between Wnt signaling and metastasis. Wnt signaling stimulates EMT and activates Snail, a transcription factor promoting metastasis [17]. Moreover, the action of Wnt ligands activates β-catenin expression (a key mediator of canonical Wnt signaling), elevates snail protein expression, and drives the EMT process of metastasis [18]. Wnt5a has been found to enhance breast cancer cell migration and invasion [19,20]. The Wnt receptor, lipoprotein receptor-related protein 6 (LRP6), is associated with cascade signaling, cell proliferation, and tumor growth [21]. Furthermore, Wnt-regulating proteins such as Naked1, Naked2, Dishevelle2 (Dvl2), and Dishevelle3 (Dvl3) are reported to be related to cancer cell metastasis [22–25]. These findings highlight the significant relationship between Wnt signaling and the EMT process in cancer cells.

Endocrine resistance arises in ER-positive breast cancer patients following antihormonal therapy. The failure of this therapy significantly increases the risk of metastasis progression. Treatment alternatives for these patients are scarce. The anticancer impact of IVM in endocrine-resistant breast cancer cells has not been reported yet. These cells have been observed to activate the human epidermal growth factor receptor 2 (HER-2) pathways and stimulate downstream signalings, such as AKT and MAPK, leading to a significant overexpression of EMT genes [26–29]. Consequently, IVM could potentially inhibit cell proliferation and metastasis in endocrine-resistant cancer cells by modulating the Wnt signaling pathway. This study is designed to uncover the anticancer effect of IVM in endocrine-resistant breast cancer cells via the Wnt signaling pathway associated with metastasis.

## Materials and methods

### Cell lines and cultures

The MCF-7 cell line was procured from the American Type Culture Collection (Virginia, USA). Tamoxifen-resistant MCF-7/LCC2 and tamoxifen- and fulvestrant-resistant MCF-7/LCC9 cells were obtained from Dr. Robert Clarke at the Lombardi Cancer Center, Georgetown University (Washington DC, USA). MCF-7/LCC2 and MCF-7/LCC9 cells were maintained in Minimum Essential Media with 5% Fetal Bovine Serum (Gibco, New York, USA). The cells were cultured at 37°C in a 5% CO_2_ and 95% humidity incubator.

### Reagents

IVM, 4-hydroxytamoxifen (4-OHT), and 3−4,5-dimethyl-2-thiazolyl-2,5-diphenyl-2H-tetrazoliumbromide (MTT) were sourced from Sigma-Aldrich (Missouri, USA). Palbociclib (PAL) was procured from Abcam (Cambridge, UK). All antibodies, including E-cadherin, N-cadherin, vimentin, snail, β-catenin, Wnt1, Wnt3a, Wnt5a/b, DDK1, LRP6, Axin1, Dvl2, Dvl3, Naked2, Naked3, and GAPDH, were purchased from Cell Signaling Technology (Massachusetts, USA).

### MTT assay

MCF-7, MCF-7/LCC2, and MCF-7/LCC9 cells were seeded at a density of 5 × 10^3^ cells per well in 96-well plates and cultured overnight. Cells were then washed and treated with IVM in a twofold dilution ranging from 3.12–50 µM for durations of 24, 48, and 72 h. A 0.02% dimethyl sulfoxide (DMSO) solution was used as a nontreatment control. A positive control for the resistant cell line was prepared using PAL in a twofold dilution from 1.56–50 µM.

For combined treatment, MCF-7/LCC2, and MCF-7/LCC9 cells were treated with IVM in 3 concentrations ranging from 3–9 µM and 4-OHT in 4 concentrations ranging from 2.5–10 µM for durations of 72 h.

Following treatment, an MTT solution (5 mg/mL) in phosphate-buffered saline (PBS) was added to each well and continuously cultured for 4 h. Formazan crystals were then dissolved with DMSO and measured by a microplate reader at 570 nm. The percentage relative to cell viability was calculated using the equation: (OD sample/OD control) × 100.

### Scratch assay

MCF-7/LCC-2 and MCF-7/LCC9 cells were seeded at densities of 1.5 × 10^6^ and 1.8 × 10^6^ cells/well into 6-well plates, respectively. The cells were cultured overnight, then wounds were scratched using a 200 µl-tip. After 24 h, the cells were treated with IC_50_ and two concentrations below IC_50_ value of IVM at 3, 6, and 9 µM for both MCF-7/LCC2 and MCF-7/LCC9 cells. 0.02% DMSO and 25 µM of PAL treatments served as the nontreatment and positive control, respectively. The scratch assays were conducted as previously described [26].

### Invasion assay

Cells in suspension were seeded at a density of 2.5 × 10^5^ cells per well in Matrigel-coated transwell inserts (Corning, USA), with 10% fetal bovine serum in the lower chamber. Concentrations at IC_50_ and two concentrations below the IC_50_ value of IVM, including 3, 6, and 9 µM were added to each well of MCF-7/LCC2 and MCF-7/LCC9 cells. 0.02% DMSO and 25 µM PAL served as nontreatment and positive controls, respectively. The upper and lower sides of the membranes were fixed with 3.7% (w/v) formaldehyde for 30 min, followed by membrane permeability with absolute methanol for 30 min, and finally stained with 0.1% (w/v) crystal violet (Sigma, USA) solution for 2 h. After each step, the cells were rinsed twice with PBS. The inhibition of invasion capacity was calculated as a relative percentage of cells invading the Matrigel-coated insert membrane compared to the untreated cells.

### Western blot analysis

MCF-7/LCC2, MCF-7/LCC9, and MCF-7 cells were exposed to IVM at concentrations of 3, 6, and 9 µM for 24 hours. 0.02% DMSO and 25 µM PAL served as nontreatment and positive controls, respectively. Cells were collected and lysed with lysis buffer for a western blot, as previously described [26]. Equal proteins were loaded and subsequently transferred to the nitrocellulose membrane. Membranes were blocked in a 5% nonfat milk solution for 1 h at room temperature and then incubated with primary antibodies in 5% bovine serum albumin in TBS-T (0.1% tween 20) buffer at 4°C overnight. The membranes were washed three times with TBS-T and then incubated with an antirabbit HRP-linked antibody in a blocking solution. Protein bands were identified. The band intensity of each protein, relative to GAPDH, was quantified using Image Studio 5.2 software (LICOR, Lincoln, USA).

### Immunofluorescence staining

MCF-7/LCC2 and MCF-7/LCC9 cells were seeded at a density of 7.5 × 10^4^ cells/well on an 8-well cell culture slide and cultured overnight. Cells were treated with either 0.018% DMSO (non-treatment) or 9 µM IVM (IVM-treatment) for 24 h. Immunofluorescent staining was performed using an immunofluorescence applications solution kit obtained from Cell Signaling Technology (Massachusetts, USA). Briefly, media were removed and cells were washed with PBS and fixed with 4% formaldehyde. The cells were washed with PBS, and blocked with blocking buffer for an hour. A β-catenin antibody was applied as the primary antibody at a 1:50 ratio in antibody dilution buffer and left at 4°C  overnight. After washing with PBS, an anti-rabbit IgG with Alexa Fluor 488 conjugate was added as the secondary antibody at a 1:600 ratio in antibody dilution buffer at room temperature for 1 hour in the dark. The cells were then washed with PBS. Coverslips were mounted on the slide using Prolong Gold Antifade with DAPI from Cell Signaling Technology. Finally, images were captured under an inverted fluorescence microscope using Nikon Camera DS-Ri2.

### Ethical consideration

This study was exempted by the Institutional Review Board of the Faculty of Medicine, Chulalongkorn University (IRB Number: 0514/65, COE No.036/2022).

### Statistical analysis

Data were presented as mean ± SEM from at least three independent experiments. Comparisons between nontreatment control and experimental groups were determined by one-way ANOVA followed by Dunnett’s test. A Student’s t-test was used to evaluate comparisons between two groups. Statistical significance was accepted at *p-value < 0.05. The statistical analysis was performed using Prism 10 software (Chicago, IL, USA).

## Results

### The inhibitory effect of IVM on cell proliferation in endocrine-resistant and ER-positive breast cancer cell lines

**Fig 1:**
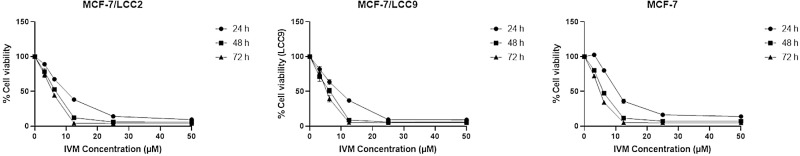
IVM inhibited the growth of endocrine-resistant breast cancer cell lines. The MTT assay measured cell viability from various IVM-treated concentrations. Cell viability was assessed in three breast cancer cell lines: MCF-7/LCC2, MCF-7/LCC9, and MCF-7 at 24, 48, and 72 h. The graphs displayed the mean ± SEM at each treated concentration compared to non-treatment (n = 3).

To assess IVM’s impact on cell viability, endocrine-resistant and ER-positive breast cancer cell lines (MCF-7/LCC2, MCF-7/LCC9, and MCF-7) were treated with varying IVM concentrations (3.12–50 µM). Cell viability percentage was determined posttreatment at 24, 48, and 72 hours. IVM reduced cell proliferation across all breast cancer cell lines at each time point (Fig 1). The half-maximal inhibitory concentration (IC_50_) values for MCF-7/LCC2, MCF-7/LCC9, and MCF-7 cells were 9.35, 9.06, and 10.14 µM respectively, after 24 h of treatment. For the 48-h treatment, IC_50_ values were 6.62, 6.35, and 6.01 µM respectively. After 72 h of treatment, IC_50_ values were 5.64, 5.43, and 4.91 µM (Table 1). Each cell line exhibited a significant IC_50_ change compared to the 24-h treatment. Notably, IC_50_ values of both endocrine-resistant cell lines were roughly equivalent to wild-type ER-positive cells at each exposure time. For IC_50_ values of PAL and 4-OHT as the positive controls in breast cancer cell lines were provided in S1 Table.

**Table 1:** Half inhibitory concentration (IC_50_) of IVM in a breast cancer cell line at different exposure times.

Cell line	Ivermectin IC_50_ (µM)		
	24 h	48 h	72 h
MCF-7/LCC2	9.35 ± 0.50	6.62 ± 0.08*	5.64 ± 0.09*
MCF-7/LCC9	9.06 ± 0.64	6.35 ± 0.45*	5.43 ± 0.17*
MCF-7	10.14 ± 0.35	6.01 ± 0.23*	4.91 ± 0.14*

*p *< 0.05* comparing to IC_50_ at 24 h posttreatment.

The data were presented as mean ± SEM (n = 3).

Combined treatments of 4-OHT  with IVM in endocrine-resistant cells to evaluate the impact of IVM on endocrine resistance were performed. The results demonstrated that combined treatment with IVM allowed for reduced concentrations of 4-OHT used to inhibit cell proliferation in all MCF-7/LCC2, MCF7-/LCC9 cells, and MCF-7 (S1A**-**S1C Figs). However, only the highest concentration of IVM at 8 µM was significantly different when compared to 4-OHT alone in MCF-7 cells (S1C Fig).

### IVM inhibited cell migration in endocrine-resistant breast cancer cell lines

**Fig 2:**
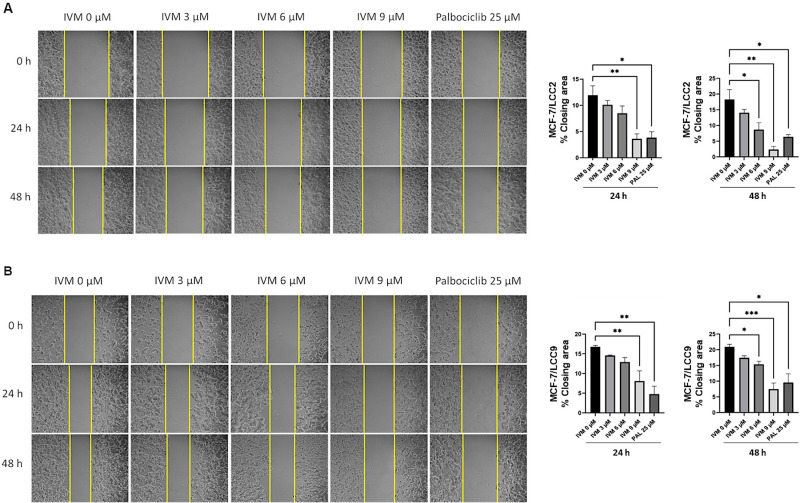
IVM inhibited cell migration in endocrine-resistant breast cancer cells. The scratch assays measured the closing area post-IVM treatment from various non-toxic concentrations and PAL treatment. The closing area of endocrine-resistant breast cancer cells: (A) MCF-7/LCC2 and (B) MCF-7/LCC9 were measured and analyzed at 24 and 48 h. The data of % closing area of MCF-7/LCC2 and MCF-7/LCC9 were presented as mean ± SEM. *p < 0.05, **p < 0.01, ***p < 0.001, ****p < 0.0001 versus the non-treatment control (n = 3).

To examine IVM’s impact on cell migration, three concentrations at IC_12.5_, IC_25_ and IC_50_ of IVM (3, 6, and 9 µM) were applied to MCF-7/LCC2 and MCF-7/LCC9 cells for 24 and 48 h posttreatment. IVM significantly curtailed wound closure in both MCF-7/LCC2 (Fig 2A) and MCF-7/LCC9 cells (Fig 2B**), (**S2 Table). IVM treatment at 9 µM (IC_50_) led to a 70% reduction in cell migration area in MCF-7/LCC2 and a 52% reduction in MCF-7/LCC9 cells at 24 hours compared to the negative control. Furthermore, at 48 h post-IVM treatment, the inhibition of migration area was even more pronounced, with reductions of 87% and 64% in MCF-7/LCC2 and MCF-7/LCC9 cells, respectively. Additionally, at a concentration of 6 µM (IC_25_), IVM also demonstrated a significant reduction in migration area at 48 hours posttreatment compared to the negative control. PAL treatment at 25 µM (IC_50_) also inhibited wound closure in both endocrine-resistant breast cancer cell lines. These findings suggest that IVM can inhibit cell migration in endocrine-resistant breast cancer cell lines.

### IVM inhibited cell invasion in endocrine-resistant breast cancer cell lines

**Fig 3:**
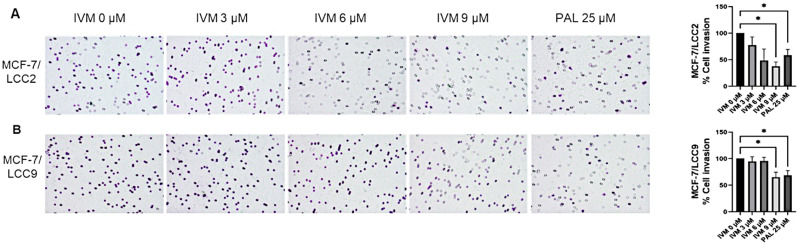
IVM inhibited the invasion of endocrine-resistant breast cancer cells. An invasion assay was conducted to quantify the invading cells in the transwell post-treatment with IVM at various non-toxic concentrations and PAL. The count of endocrine-resistant breast cancer cells (A) MCF-7/LCC2 and (B) MCF-7/LCC9 was taken 24 h post-treatment. The data representing %cell invasion relative to the non-treatment control for MCF-7/LCC2 and MCF-7/LCC9 were displayed as mean ± SEM. *p < 0.05 when compared to the non-treatment control (n = 3).

To assess IVM’s impact on cell invasion, MCF-7/LCC2 **(**Fig 3A) and MCF-7/LCC9 (Fig 3B) cells were exposed to IVM at three concentrations at IC_50_ and two concentrations below IC_50_ value for 24 h on Matrigel-coated transwell inserts (S3 Table). IVM significantly curtailed cell invasion by 62% and 35% at 9 µM (IC_50_) in MCF-7/LCC2 and MCF-7/LCC9 cells, respectively, compared to negative controls. PAL treatment at 25 µM (IC_50_ concentration) also significantly reduced cell invasion in both endocrine-resistant breast cancer cell lines. These results suggest that IVM mitigates metastasis in endocrine-resistant cell lines by reducing their invasive capability.

### IVM reduced EMT-associated proteins in endocrine-resistant breast cancer cell lines

**Fig 4:**
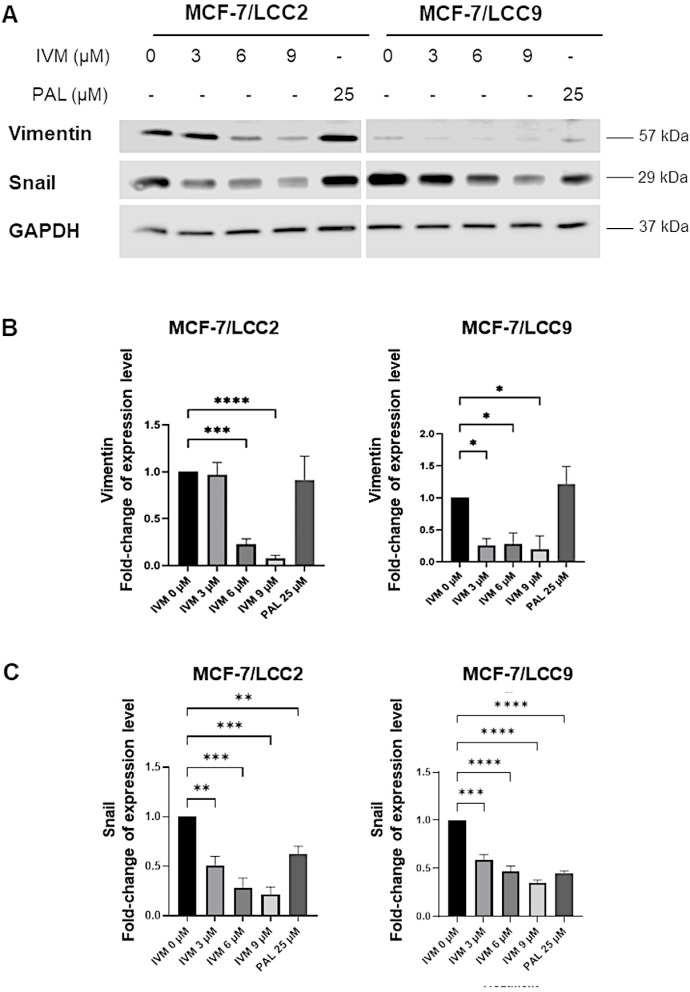
IVM impact on EMT markers in breast cancer cell lines: MCF-7/LCC2 and MCF-7/LCC9. (A) Protein expression was measured by western blot after treating with various concentrations of IVM for 24 h. The graphs depicted the inhibitory effect of IVM on EMT-associated proteins, including (B) Vimentin, and (C) Snail. The data were presented as mean ± SEM. *p < 0.05, **p < 0.01, ***p < 0.001, ****p < 0.0001 when compared to the non-treatment control (n = 3).

The impact of IVM on cell invasion was assessed by examining the expression of EMT-related proteins in MCF-7/LCC2 and MCF-7/LCC9 cells (Fig 4A) following a 24-h treatment with IVM at three concentrations at IC_50_ and two concentrations below IC_50_ value. IVM notably reduced the expression of vimentin and snail in both MCF-7/LCC2 and MCF-7/LCC9 cells (Figs 4B **and** 4C). The expression of Snail also decreased with PAL treatment in both endocrine-resistant breast cancer cell lines (Fig 4C). However, both endocrine-resistant breast cancer cell lines showed no significant alterations in N-cadherin expression (S2A **and** S2B Figs). For MCF-7 cells, the bands of Vimentin could not be detected. In addition, IVM inhibited Snail, but the inhibitory result was not concentration-dependent manner (S3A **and** S3B Figs). Therefore, IVM mitigates EMT by suppressing transcription factors implicated in cell invasion.

### IVM interfered with Wnt signaling pathway in endocrine-resistant breast cancer cell lines

**Fig 5:**
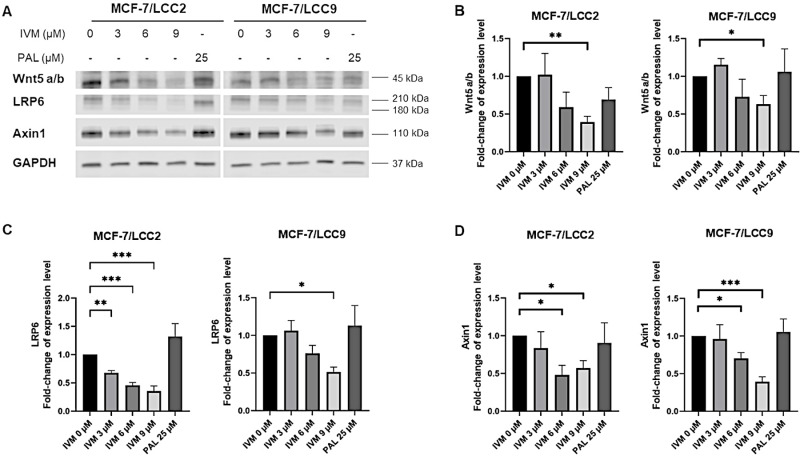
The influence of IVM on Wnt signaling proteins in endocrine-resistant breast cancer cell lines: MCF-7/LCC2 and MCF-7/LCC9. (A) Protein expression at various concentrations of IVM treatment for 24 h. The graphs displayed a fold-change in protein expression level due to the impact of IVM on (B) Wnt5a/b, (C) LRP6, and (D) Axin1. The data (n = 3) were presented as mean ± SEM. *p < 0.05, **p < 0.01, ***p < 0.001 compared to the non-treatment control (n = 3).

To examine IVM’s effects on the Wnt signaling pathway, MCF-7/LCC2 and MCF-7/LCC9 cells were treated with IVM at three concentrations at IC_50_ and two concentrations below IC_50_ value for 24 h, and the expression of proteins associated with Wnt signaling was observed (Fig 5A). Determination of Wnt ligands showed that Wnt5a/b expression was significantly reduced at 9 µM (IC_50_) of IVM treatment (Fig 5B). Interestingly, IVM also decreased the expression of LRP6 (Fig 5C) and Axin1 (Fig 5D), proteins associated with activation at the Wnt receptor, in both endocrine-resistant breast cancer cell lines. The expression of β-catenin remained unchanged among both endocrine-resistant breast cancer cells treated with IVM (S2A **and** S2C Figs). Other proteins correlated with the Wnt signaling pathway, including Naked1, Naked2, Dvl2, and Dvl3, revealed no change in expression after IVM treatment (S2A **and** S2D**-**2G Figs). PAL treatment did not affect the Wnt signaling pathway in both endocrine-resistant breast cancer cell lines. These findings indicate that IVM mainly affects the Wnt receptor and certain Wnt ligands in endocrine-resistant breast cancer cells. The experiments to assess the inhibitory effect of IVM on Wnt signaling in MCF-7 wild-type cells were conducted. However, the Western Blot bands of Wnt5a/b could not be detected. IVM significantly inhibited only LRP6 at 9 µM which is the highest concentration (S4A**-**S4C Figs) and had no effect on other proteins of Wnt signaling in MCF-7 wild-type cells (S5A**-**S5G Figs).

### IVM did not alter the localization of β-catenin in endocrine-resistant breast cancer cell lines

**Fig 6:**
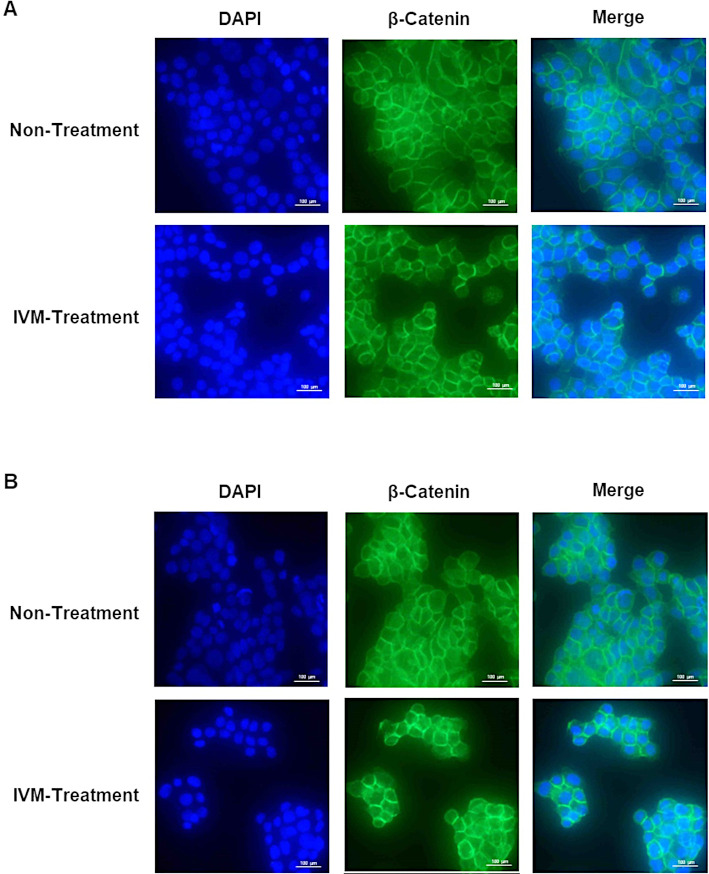
Effect of IVM on the expression and localization of β-catenin in endocrine-resistance cell lines. Inverted fluorescence microscopy images show the fluorescent staining antibody post-treatment with IVM at 9 µM (IVM-treatment) and control (non-treatment) for 24 h in (A) MCF-7/LCC2 and (B) MCF-7LCC9. Cell nuclei stained with DAPI. Anti-β-catenin was applied at a 1:50 dilution and is represented in green. Scale bares = 100 µm.

To further investigate the mechanism of IVM in Wnt signaling pathway, the effect of IVM on the localization of β-catenin was examined by immunofluorescence assay. MCF-7/LCC2 and MCF-7/LCC9 cells were treated with IVM at IC_50_ values for 24 h. The localization of β-catenin mostly remained at the cell membranes after the treatment of IVM in both MCF-7/LCC2 cells (Fig 6A) and MCF-7/LCC9 cells (Fig 6B). Thus, IVM did not affect the localization of β-catenin in endocrine-resistant breast cancer cells.

## Discussion

IVM, a clinical drug used in treating various parasitic diseases, has shown potential effects on different cancer types, including breast cancers [13]. A published study in Cancer Research assessed the impact of IVM on several breast cancer cell lines and a non-tumorigenic breast epithelial cell line (MCF-10A). The results indicated that IVM significantly decreased cell viability in breast cancer cell lines in a concentration-dependent manner; the IC₅₀ value of MCF-10A cells was much higher, suggesting a lower sensitivity of non-tumorigenic cells to IVM compared to cancer cells. This finding suggested that non-tumorigenic cells are less sensitive to IVM, supporting its potential selectivity toward cancer cells [6]. Research has demonstrated IVM’s impact on breast cancer by arresting the cell cycle [13,30–33], inducing autophagy [6], and stimulating inflammation [6,12,34]. Additionally, IVM has been found to inhibit the oncogenic [35] and cancer-resistant proteins [36]. Interestingly, IVM can reverse the effects of drug treatments in drug-resistant breast cancer cells, such as tamoxifen and doxorubicin [11,13,37]. This evidence could be applied to IVM in treating endocrine-resistant breast cancers.

Our research findings indicate that IVM has antiproliferative effects on endocrine-resistant breast cancer cells, with IC_50_ values in the micromolar range. The observation showed that the IC_50_ values of endocrine-resistant breast cancer cells, MCF-7/LCC2 and MCF-7/LCC9, are in a similar range to that of wild-type MCF-7 at every exposure time, with IC_50_ under 10 µM. Thus, the potency of IVM in endocrine-resistant breast cancers is similar to wild-type cancer cells. Previous studies have reported that the IC_50_ range of MCF-7 cells is similar to our IVM treatment result [6]. Additionally, when IVM-treated MCF-7 using an ethanol vehicle was treated for 72 h, the IC_50_ was found to be three times lower than our observation using a DMSO vehicle [13]. This data suggests that the antiproliferative effect of IVM may vary depending on the choice of the vehicle solvent, while the efficiency is consistent across experiments.

In addition, co-treatment with IVM allowed for reduced concentrations of tamoxifen while maintaining efficacy, suggesting that IVM may help overcome resistance to these therapies. Further investigation into the underlying mechanisms is ongoing and will be addressed in future work.

Metastasis is a progression of breast cancer cells, particularly in drug-resistant cancer. Clinical studies have shown that different breast cancer subtypes, based on hormonal receptor (HR) and human epidermal growth factor receptor (HER-2) expression, exhibit varying metastasis rates [38]. Subtypes with HR overexpression and low HER-2 expression had the lowest metastasis rate, while those with low HR expression and HER-2 overexpression had the highest rate. Our previous report indicated that endocrine-resistant breast cancer cell lines MCF-7/LCC2 and MCF-7/LCC9 overexpress HER-2 [27], suggesting that endocrine-resistant breast cancers are more aggressive than the wild-type MCF-7 cells. IVM has been shown to have antimigration effects in cervical carcinoma (HeLa) migration [32] and breast cancer (wild-type MCF-7) cells [14] and exhibits an antiinvasive effect on wild-type MCF-7 breast cancer cells [14]. Our results demonstrated that IVM effectively reduces both migration and invasion, indicating its potential to inhibit the motility of endocrine-resistant breast cancer cells, potentially preventing disease progression. In the metastasis process, our previous report shows that endocrine-resistant breast cancer (MCF-7/LCC9) overexpressed EMT genes, including vimentin and snail, and had lower E-cadherin expression compared to wild-type (MCF-7) cells [26]. Suppression of Snail expression is decreased EMT (invasion) in MCF-7/LCC9 cells [39]. Our results demonstrated that IVM significantly decreased vimentin and snail expression in a concentration-dependent manner in both endocrine-resistant cell lines, MCF-7/LCC2 and MCF-7/LCC9. Additionally, IVM was able to increase E-cadherin expression in triple-negative breast cancer MDA-MB-231 cells [11]. This evidence suggests that IVM has the potential to inhibit metastasis of endocrine-resistant breast cancer cells.

Wnt signaling plays a role in cell proliferation and metastasis in breast cancer [15]. The correlation of the Wnt signaling pathway in EMT has shown that Snail activation is related to EMT [17]. Wnt ligands, including Wnt1, Wnt3, Wnt5a, and Wnt5b, promote EMT progression [18,20,40,41]. Therefore, Wnt signaling is a targeted pathway for therapeutic approaches to breast cancer metastasis [42]. Numerous studies have indicated that the expression of Wnt ligands is linked to metastasis. An increase in Wnt1 ligand upregulated snail expression, driving EMT [18]. Wnt3a was found to promote EMT in colon cancer [43]. A decrease in Wnt5a and Wnt5b suppressed invasion in breast cancer [44,45]. Consistent with our results, IVM reduced the level of Wnt5a/b in endocrine-resistant breast cancer cells. Additionally, in triple-negative breast cancer, Wnt5a was reduced after selamectin treatment [11]. Moreover, the regulatory proteins of Wnt signaling, Naked1 and Naked2, have antagonist effects on Wnt signaling of cell differentiation [22]. A decrease in Naked1 expression increased the invasive effect on lung cancer cells [23]. Naked2 was found to suppress breast cancer proliferation via Wnt signaling in breast cancers [46]. Our findings demonstrated that the effect of IVM did not alter the expression of Naked1 and Naked2, suggesting that IVM inhibits the action of Wnt5a/b ligands without modifying the expression of negative regulatory proteins.

Regarding Wnt receptors, we observed that IVM reduced the expression of LRP6 in endocrine-resistant breast cancers. This aligns with results showing that silencing LRP6 decreased Wnt signaling, cell proliferation, and tumor growth in breast cancer [21]. This evidence supports the potential of LRP6 as a therapeutic target for breast cancer. Downstream proteins in the canonical Wnt signaling pathway of breast cancer, including adenomatous polyposis coli, Wilms tumor gene on X chromosome (WTX), protein phosphatase 2A (PP2A), Dvl, and axin, are recruited into the β-Catenin deconstruction complex [15]. In colorectal cancer, upregulation of Dvl2 and Dvl3, enhancing Wnt signaling, promotes metastasis progression [24,25]. Our results showed that IVM does not affect Dvl2 and Dvl3 proteins, indicating that IVM does not interfere with the alteration of Dvls expression in the deconstruction complex. Axin1, a main scaffold protein for the β-Catenin destruction complex in the Wnt signaling pathway, plays a role in both activation and inactivation of the pathway [47]. Increasing Axin1 in the deconstruction complex degrades β-Catenin expression, inhibiting the Wnt signaling cascade [48]. Moreover, our results showed that IVM also inhibited Snail and LRP6 in ER-positive wild-type breast cancer cells, although the effect was less pronounced compared to hormone-resistant cells. Wnt signaling pathway is upregulated in an *in vitro* model of acquired tamoxifen-resistant breast cancer, suggesting its involvement in resistance mechanisms [49]. This differential effect may be attributed to the overexpression of mesenchymal marker and Wnt signaling pathways in endocrine-resistant cells.

PAL, a selective CDK4/6 inhibitor, is clinically approved for the treatment of endocrine-resistant ER-positive, HER2-negative breast cancer. In this study, PAL was used as a positive control for EMT inhibition due to its well-documented antiproliferative effects and ability to suppress EMT-related phenotypes in breast cancer models. Several studies have shown that PAL reduces expression of mesenchymal markers such as Vimentin and Snail while restoring epithelial markers like E-cadherin, thereby inhibiting migration and invasion of breast cancer cells [50,51]. These effects have been linked to the modulation of signaling pathways such as c-Jun/COX-2 and AKT/mTOR, suggesting that PAL can influence EMT beyond its cell cycle regulatory role. PAL, when combined with olaparib, inhibited Wnt/β-catenin signaling—specifically by reducing β-catenin Ser675 phosphorylation and MYC expression—thereby reversing EMT and overcoming olaparib resistance in triple-negative breast cancer [52]. However, in our study, PAL did not significantly inhibit mesenchymal markers or Wnt-related mediators when compared to IVM.

β-catenin, the key mediator protein driving the Wnt signaling pathway in breast cancer, accumulates highly in cancer cells, particularly in the nucleus, leading to the activation of Wnt signaling in breast cancer [53,54]. Phosphorylated β-catenin by the destruction complex leads to β-catenin degradation, resulting in the inhibition of pancreatic cancer cell proliferation [48]. Contrary to our result, the effect of IVM has suppressed Axin1 with no alteration in β-catenin level and localization. Therefore, our studies proposed the effect of IVM in inhibiting the initiation of the Wnt signaling pathway by downregulating Wnt5a/b ligands expression, leading to a decrease in LRP6 expression. This reduction in Wnt signaling is associated with decreased EMT by suppressing vimentin and snail expression in endocrine-resistant breast cancers. A limitation of this study is that the observed changes in Wnt5a/b ligand levels and LRP6 expression may represent the pharmacological effects of IVM, rather than direct evidence of Wnt pathway involvement. To address this, future mechanistic studies are planned using specific Wnt inhibitors or siRNA targeting key Wnt components to clarify the precise role of this pathway in mediating IVM’s effects.

## Conclusion

IVM exhibits strong antiproliferative and antiinvasive properties in endocrine-resistant‍ breast cancer cells. The findings propose the mechanisms of IVM in suppressing EMT markers and the Wnt signaling receptor. Consequently, our studies offer initial results and Underlying mechanisms that support the potential repurposing of IVM for treating patients with endocrine-resistant breast cancer.

### Graphical Abstract

Ivermectin can suppress the Wnt signaling pathway, which is linked to the epithelial-to-mesenchymal transition (EMT) involved in the metastasis of endocrine-resistant breast cancer cells.

## Supporting information

S1 Fig Combined treatment of IVM and 4-OHT further reduced cell viability in endocrine-resistant breast cancer cell lines: MCF-7/LCC2 and MCF-7/LCC9. The MTT assay measured cell viability from various IVM and 4-OHT-treated concentrations. Cell viability was assessed in three breast cancer cell lines: (A) MCF-7/LCC2, (B) MCF-7/LCC9, and (C) MCF-7 at 72 h. The graphs displayed the mean ± SEM at each treated concentration. **p < 0.01, ***p < 0.001, ****p < 0.0001 compared to non-treatment control by Two-Way ANOVA Analysis (n = 3).
(DOCX)

S2 Fig IVM impact on other EMT markers and Wnt signaling proteins in endocrine-resistant breast cancer cell lines: MCF-7/LCC2 and MCF-7/LCC9. (A) Protein expression at various concentrations of IVM treatment for 24 h. The graphs displayed a fold-change in protein expression level due to the impact of IVM on (B) N-Cadherin, (C) β-Catenin, (D) Naked1, (E) Naked2, (F) Dvl2, and (G) Dvl3. The data (n = 3) were presented as mean ± SEM. The fold change was compared to the non-treatment control (n = 3).
(DOCX)

S3 Fig IVM impact on EMT markers in ER-positive breast cancer cell line: MCF-7. (A) Protein expression was measured by western blot after treating with various concentrations of IVM for 24 h. The graphs depicted the inhibitory effect of IVM on EMT-associated proteins, including (B) Snail. The data were presented as mean ± SEM. **p < 0.01, ***p < 0.001 when compared to the non-treatment control (n = 3).
(DOCX)

S4 Fig The effect of IVM on Wnt signaling in ER-positive breast cancer cell line: MCF-7. (A) Protein expression at various concentrations of IVM treatment for 24 h. The graphs displayed a fold-change in protein expression level due to the impact of IVM on (B) LRP6, and (C) Axin1. The data (n = 3) were presented as mean ± SEM. *p < 0.05 compared to the non-treatment control (n = 3).
(DOCX)

S5 Fig The effect of IVM on other EMT markers and Wnt signaling proteins in ER positive breast cancer cell line: MCF-7. (A) Protein expression at various concentrations of IVM treatment for 24 h. The graphs displayed a fold-change in protein expression level due to the impact of IVM on (B) N-Cadherin, (C) β-Catenin, (D) Naked1, (E) Naked2, (F) Dvl2, and (G) Dvl3. The data (n = 3) were presented as mean ± SEM.
(DOCX)

S1 Table Half inhibitory concentration (IC_50_) of drug controls including PAL and 4-OHT in breast cancer cell line. The data (N = 3) were shown as mean ± SEM.
(DOCX)

S2 Table IVM inhibited cell migration of endocrine-resistant breast cancer cell lines. The closing area in the scratch assay was analyzed after IVM treatment in various non-toxic concentrations at 24 and 48 h. The data (N = 3) were shown as mean of %Closing area compared with the area at 0 hours of treatment ± SEM.
(DOCX)

S3 Table IVM inhibited cell invasion of endocrine-resistant breast cancer cell lines. The Matrigel-based invasion cell assay was analyzed after IVM treatment in various non-toxic concentrations at 24 h. The data (N = 3) were shown as mean of %Inasion cells compared with non-treatment control ± SEM.
(DOCX)

S1 File Original uncropped images of all blots. (DOCX)

S2 File Raw data and statistical analysis of all figures. (DOCX)
