# Supplementary figures and images for: Ivermectin inhibits epithelial-to-mesenchymal transition via Wnt signaling in endocrine-resistant breast cancer cells
Source: PLoS One. 2025 Jun 26;20(6):e0326742. doi: 10.1371/journal.pone.0326742 (PMC12200854; doi:10.1371/journal.pone.0326742)

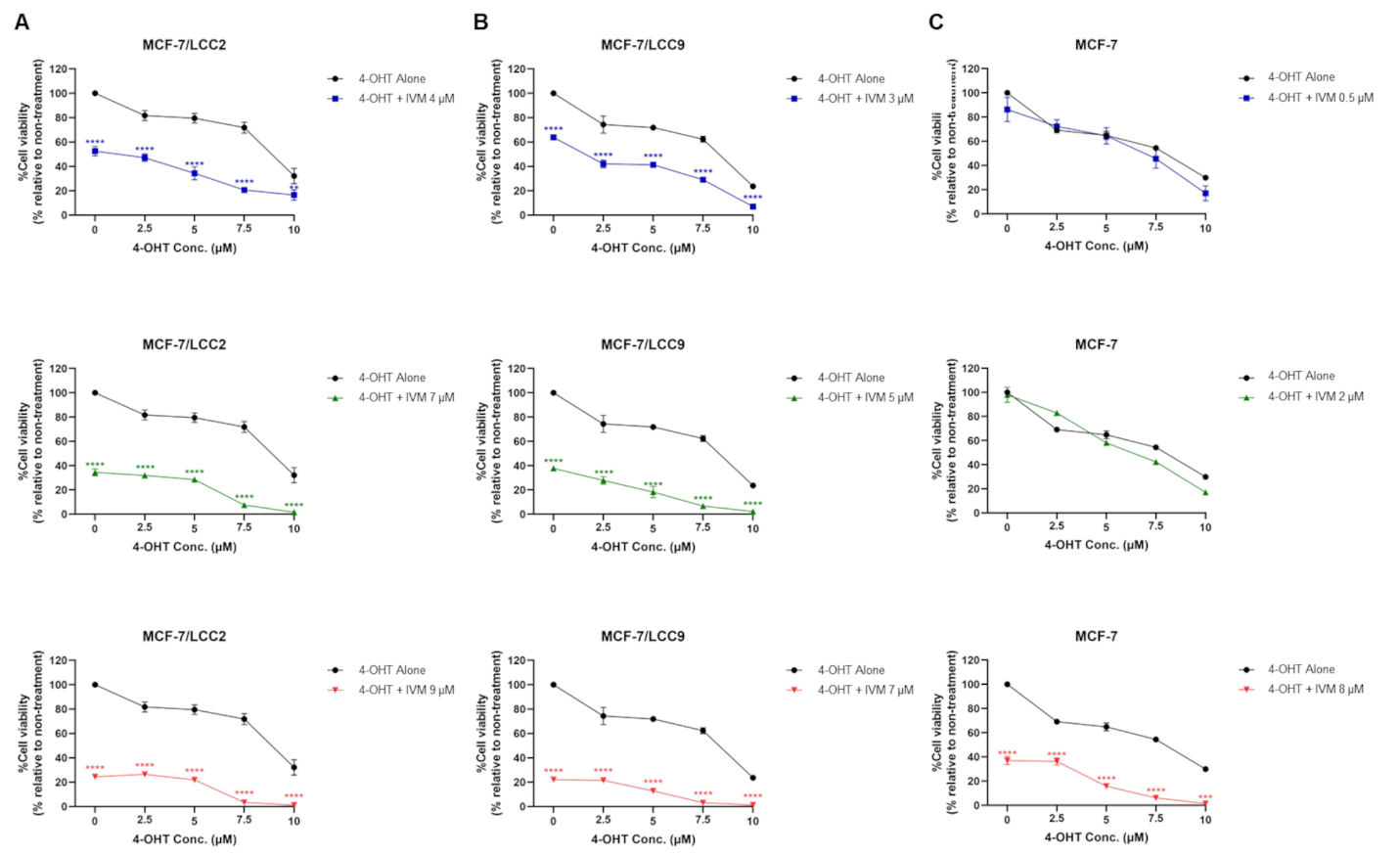


**S1 Fig**

Supplement: S1 Fig — The MTT assay measured cell viability from various IVM and 4-OHT-treated concentrations. Cell viability was assessed in three breast cancer cell lines: (A) MCF-7/LCC2, (B) MCF-7/LCC9, and (C) MCF-7 at 72 h. The graphs displayed the mean ± SEM at each treated concentration. **p < 0.01, ***p < 0.001, ****p < 0.0001 compared to non-treatment control by Two-Way ANOVA Analysis (n = 3). (DOCX) [file pone.0326742.s001.docx]

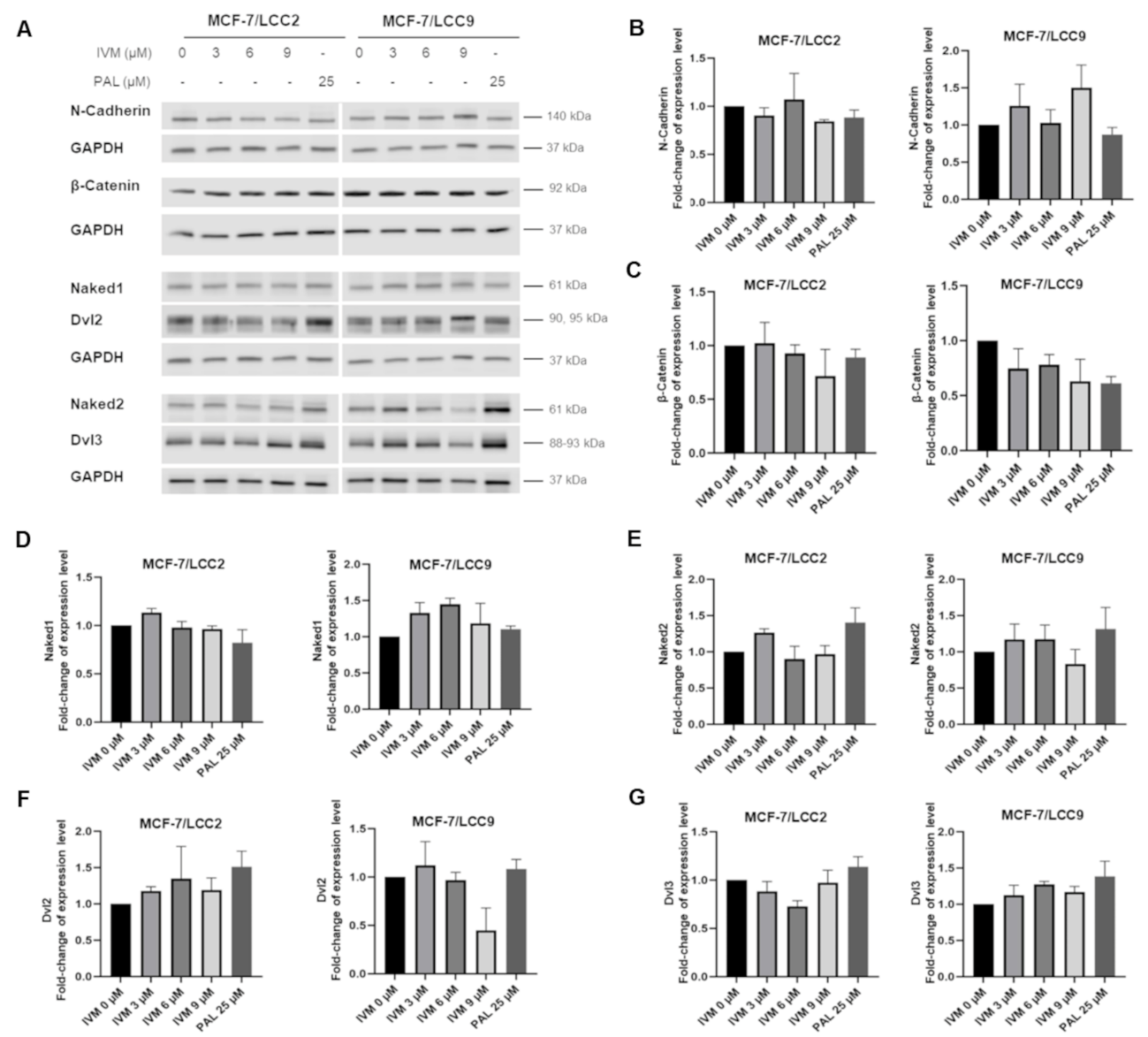


**S2 Fig**

Supplement: S2 Fig — (A) Protein expression at various concentrations of IVM treatment for 24 h. The graphs displayed a fold-change in protein expression level due to the impact of IVM on (B) N-Cadherin, (C) β-Catenin, (D) Naked1, (E) Naked2, (F) Dvl2, and (G) Dvl3. The data (n = 3) were presented as mean ± SEM. The fold change was compared to the non-treatment control (n = 3). (DOCX) [file pone.0326742.s002.docx]

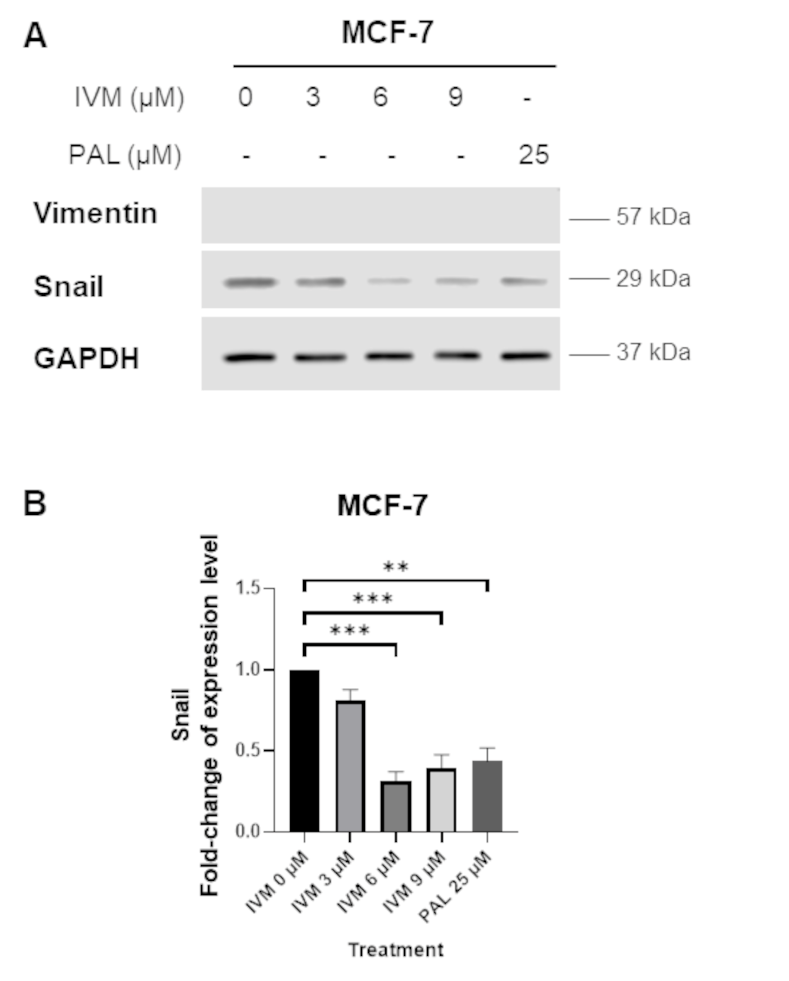


**S3 Fig**

Supplement: S3 Fig — (A) Protein expression was measured by western blot after treating with various concentrations of IVM for 24 h. The graphs depicted the inhibitory effect of IVM on EMT-associated proteins, including (B) Snail. The data were presented as mean ± SEM. **p < 0.01, ***p < 0.001 when compared to the non-treatment control (n = 3). (DOCX) [file pone.0326742.s003.docx]

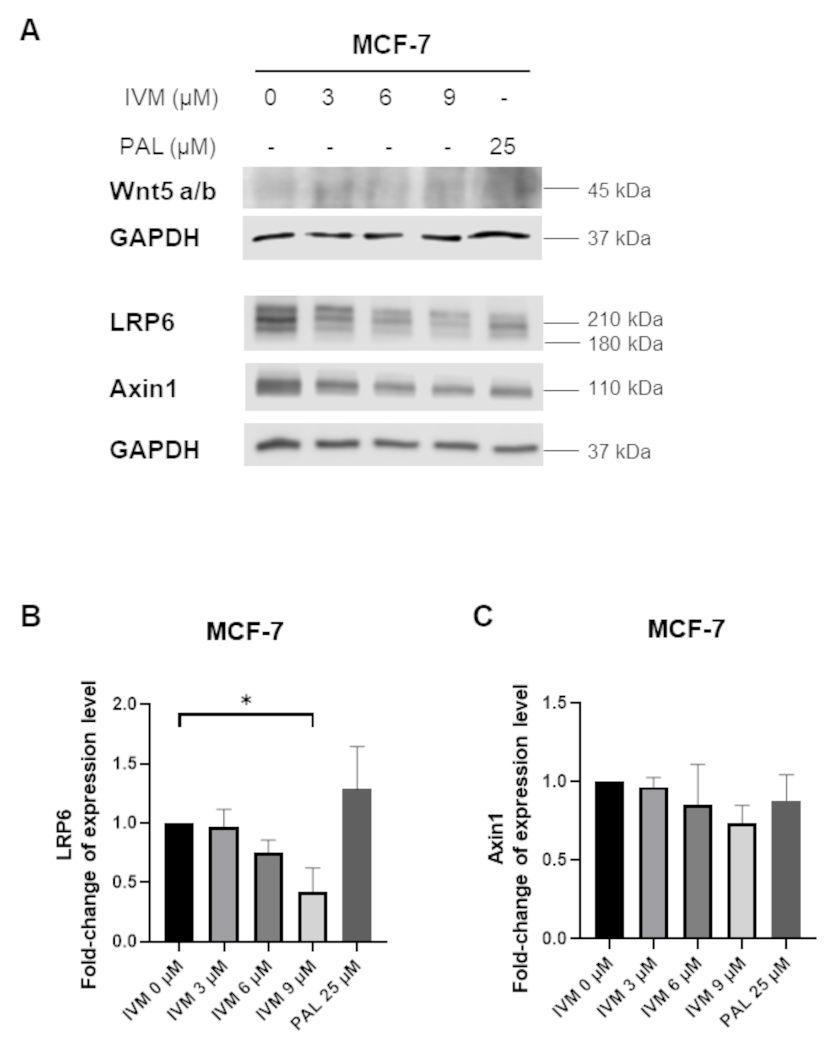


**S4 Fig**

Supplement: S4 Fig — (A) Protein expression at various concentrations of IVM treatment for 24 h. The graphs displayed a fold-change in protein expression level due to the impact of IVM on (B) LRP6, and (C) Axin1. The data (n = 3) were presented as mean ± SEM. *p < 0.05 compared to the non-treatment control (n = 3). (DOCX) [file pone.0326742.s004.docx]

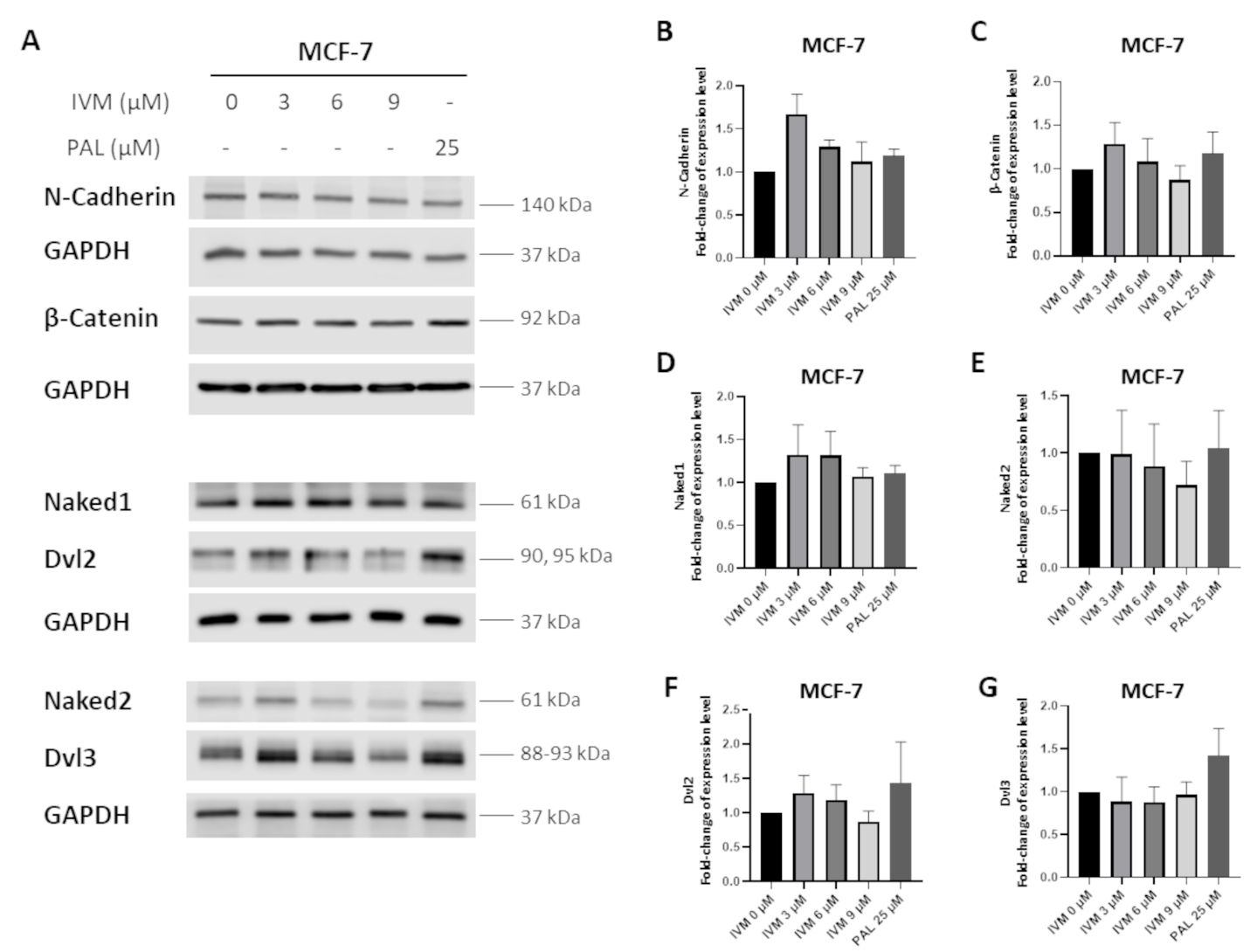


**S5 Fig**

Supplement: S5 Fig — (A) Protein expression at various concentrations of IVM treatment for 24 h. The graphs displayed a fold-change in protein expression level due to the impact of IVM on (B) N-Cadherin, (C) β-Catenin, (D) Naked1, (E) Naked2, (F) Dvl2, and (G) Dvl3. The data (n = 3) were presented as mean ± SEM. (DOCX) [file pone.0326742.s005.docx]
