# Supplementary material for: Ivermectin inhibits epithelial-to-mesenchymal transition via Wnt signaling in endocrine-resistant breast cancer cells
Source: PLoS One. 2025 Jun 26;20(6):e0326742. doi: 10.1371/journal.pone.0326742 (PMC12200854; doi:10.1371/journal.pone.0326742)
Supplement: S1 Table — The data (N = 3) were shown as mean ± SEM. (DOCX) [file pone.0326742.s006.docx]

**S1 Table.**

| **Cell line** | **Palbociclib IC_50_ (µM)** | | | **4-OHT IC_50_ (µM)** |
| --- | --- | --- | --- | --- |
|  | **24 h** | **48 h** | **72 h** | **24 h** |
| MCF-7/LCC2 | 24.02 ± 3.67 | 19.85 ± 3.18 | 17.71 ± 2.52 | 11.62 ± 0.12 |
| MCF-7/LCC9 | 25.40 ± 1.71 | 21.46 ± 2.16 | 15.85 ± 2.69 | 10.65 ± 0.91 |
| MCF-7 | 13.07 ± 0.27 | 10.60 ± 0.79 | 9.05 ± 0.46 | 6.31 ± 0.53 |
|  | | | | |
