# Supplementary material for: Ivermectin inhibits epithelial-to-mesenchymal transition via Wnt signaling in endocrine-resistant breast cancer cells
Source: PLoS One. 2025 Jun 26;20(6):e0326742. doi: 10.1371/journal.pone.0326742 (PMC12200854; doi:10.1371/journal.pone.0326742)
Supplement: S2 Table — The closing area in the scratch assay was analyzed after IVM treatment in various non-toxic concentrations at 24 and 48 h. The data (N = 3) were shown as mean of %Closing area compared with the area at 0 hours of treatment ± SEM. (DOCX) [file pone.0326742.s007.docx]

**S2 Table.**

| **Cell line** | **Time** | **%Closing area relative to the area at 0 hour of treatment** | | | | | | |
| --- | --- | --- | --- | --- | --- | --- | --- | --- |
|  |  | **Ivermectin** | | | | | | **Palbociclib** |
|  |  | **0 µM** | **3 µM** | | **6 µM** | | **9 µM** | **25 µM** |
| MCF-7/LCC2 | 24 h | 11.94 ± 0.98 | 10.11 ± 0.45 | | 8.48 ± 0.77 | | 3.6 ± 0.52 | 3.81 ± 0.63 |
|  | 48 h | 18.28 ± 1.71 | 14.06 ± 0.58 | | 8.67 ± 1.20 | | 2.32 ± 0.55 | 6.36 ± 0.41 |
| MCF-7/LCC9 | 24 h | 16.74 ± 0.22 | 14.57 ± 0.08 | | 12.93 ± 0.62 | | 8.08 ± 1.42 | 4.75 ± 1.11 |
|  | 48 h | 20.9 ± 0.48 | 17.42 ± 0.36 | | 15.35 ± 0.53 | | 7.47 ± 1.05 | 9.53 ± 1.54 |
|  | | | |  | |  |  |  |
