# Supplementary material for: Ivermectin inhibits epithelial-to-mesenchymal transition via Wnt signaling in endocrine-resistant breast cancer cells
Source: PLoS One. 2025 Jun 26;20(6):e0326742. doi: 10.1371/journal.pone.0326742 (PMC12200854; doi:10.1371/journal.pone.0326742)
Supplement: S3 Table — The Matrigel-based invasion cell assay was analyzed after IVM treatment in various non-toxic concentrations at 24 h. The data (N = 3) were shown as mean of %Inasion cells compared with non-treatment control ± SEM. (DOCX) [file pone.0326742.s008.docx]

**S3 Table.**

|  | **% Invasion cells relative to non-treatment** | | | |  |
| --- | --- | --- | --- | --- | --- |
| **Cell line** | **Ivermectin** | | | | **Palbociclib** |
|  | **0 µM** | **3 µM** | **6 µM** | **9 µM** | **25 µM** |
| MCF-7/LCC2 | 100 ± 0.00 | 77.67 ± 15.34 | 48.67 ± 21.50 | 37.67 ± 8.19 | 58.67 ± 10.73 |
| MCF-7/LCC9 | 100 ± 0.00 | 94.67 ± 8.88 | 95.67 ± 6.77 | 65.00 ± 9.61 | 68.67 ± 8.88 |
|  | | | | | |
