# Supplementary material for: Ivermectin inhibits epithelial-to-mesenchymal transition via Wnt signaling in endocrine-resistant breast cancer cells
Source: PLoS One. 2025 Jun 26;20(6):e0326742. doi: 10.1371/journal.pone.0326742 (PMC12200854; doi:10.1371/journal.pone.0326742)

**Supplementary Data**

**Western blot detection (Raw Images)**

**Figure 4B, 4C**: MCF-7/LCC2


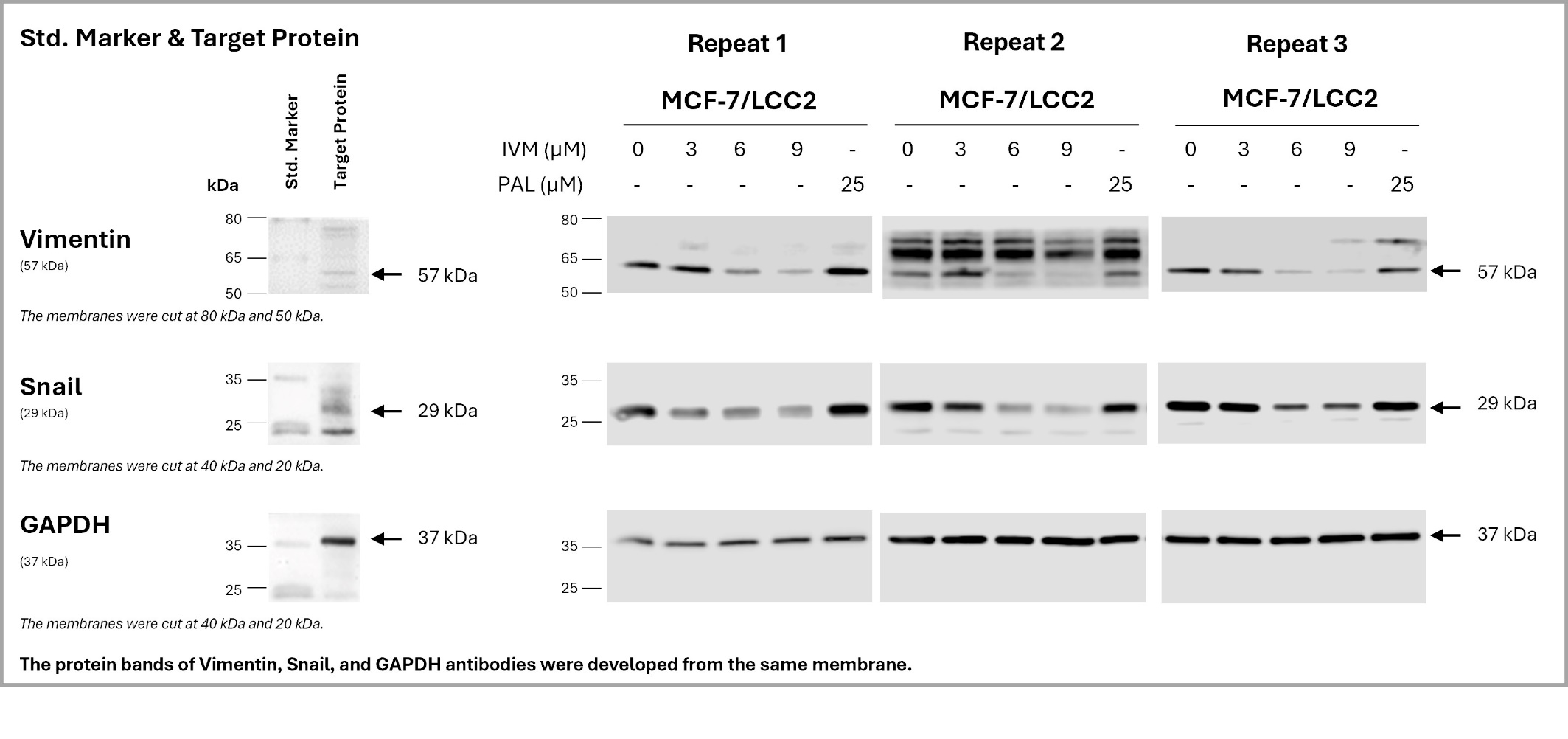


**Figure 4B, 4C**: MCF-7/LCC9


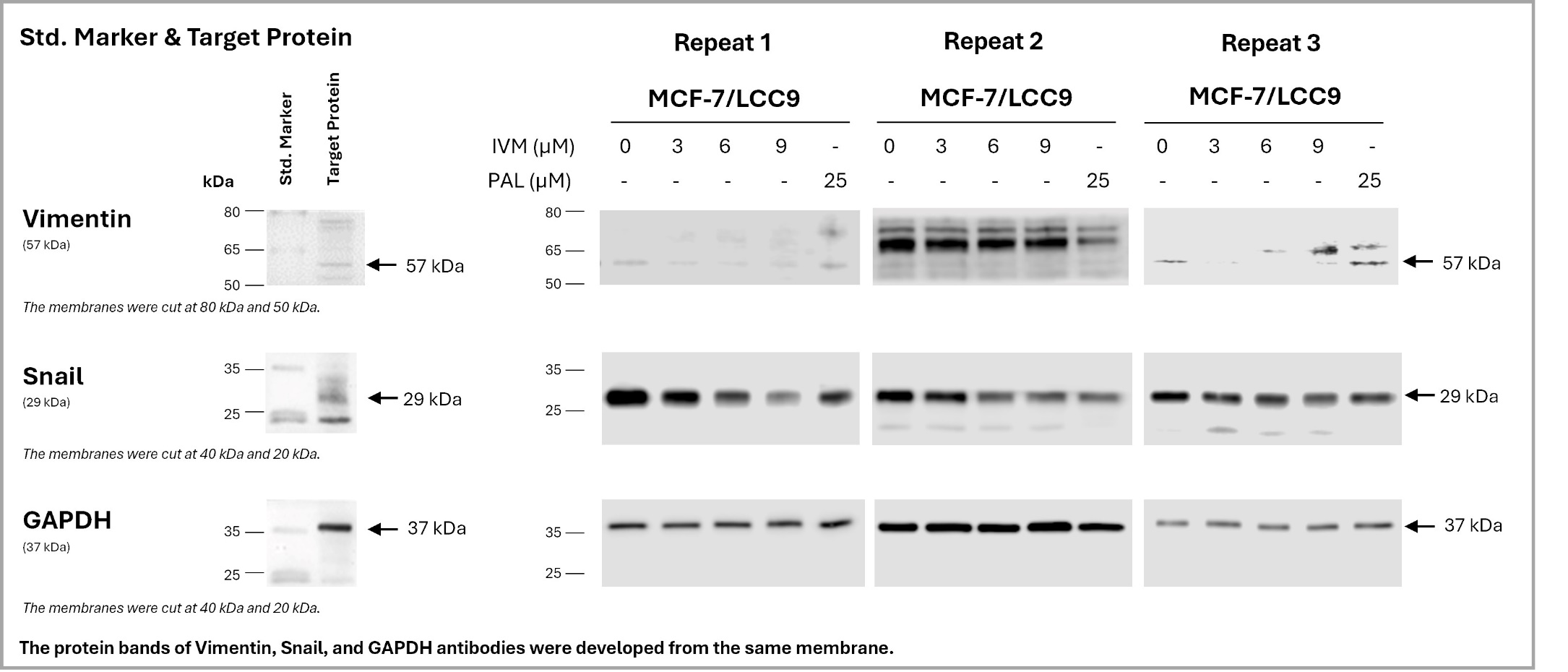


**Figure 5B, 5C**: MCF-7/LCC2


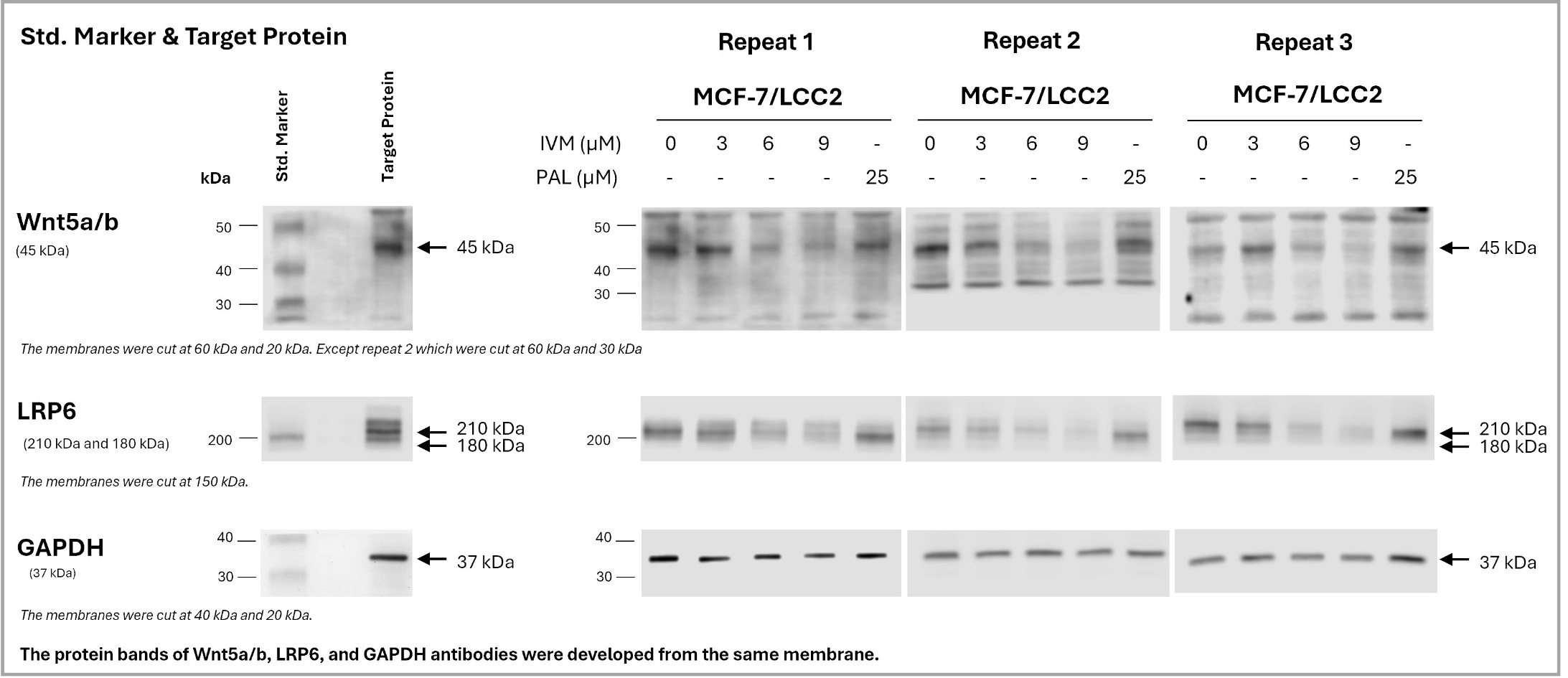


**Figure 5D**: MCF-7/LCC2


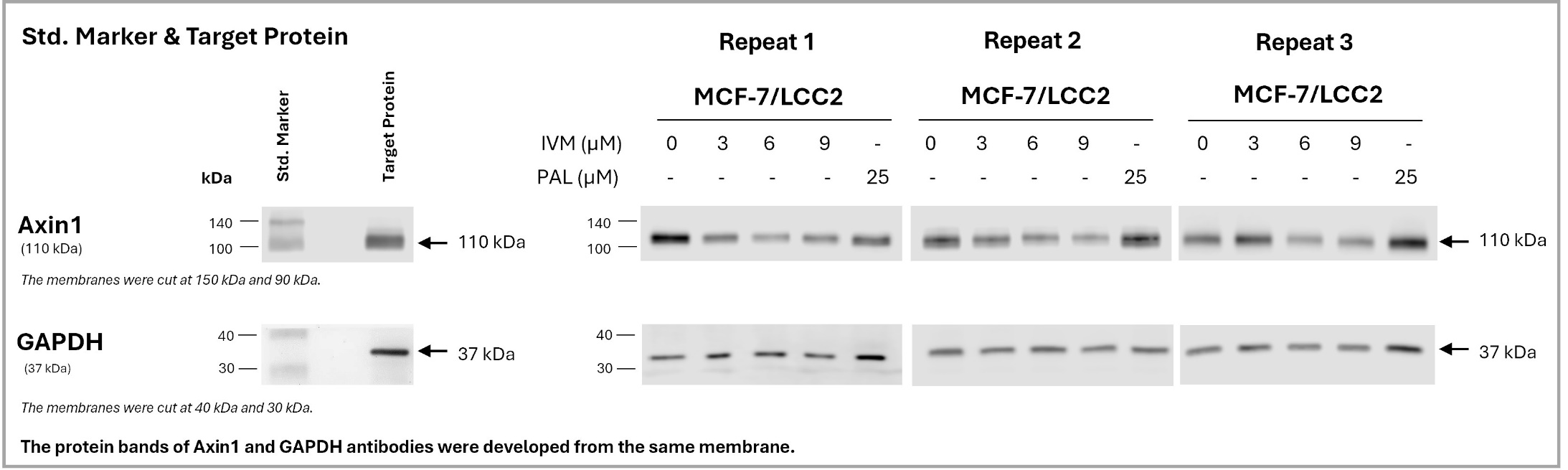


**Figure 5B, 5C, 5D**: MCF-7/LCC9


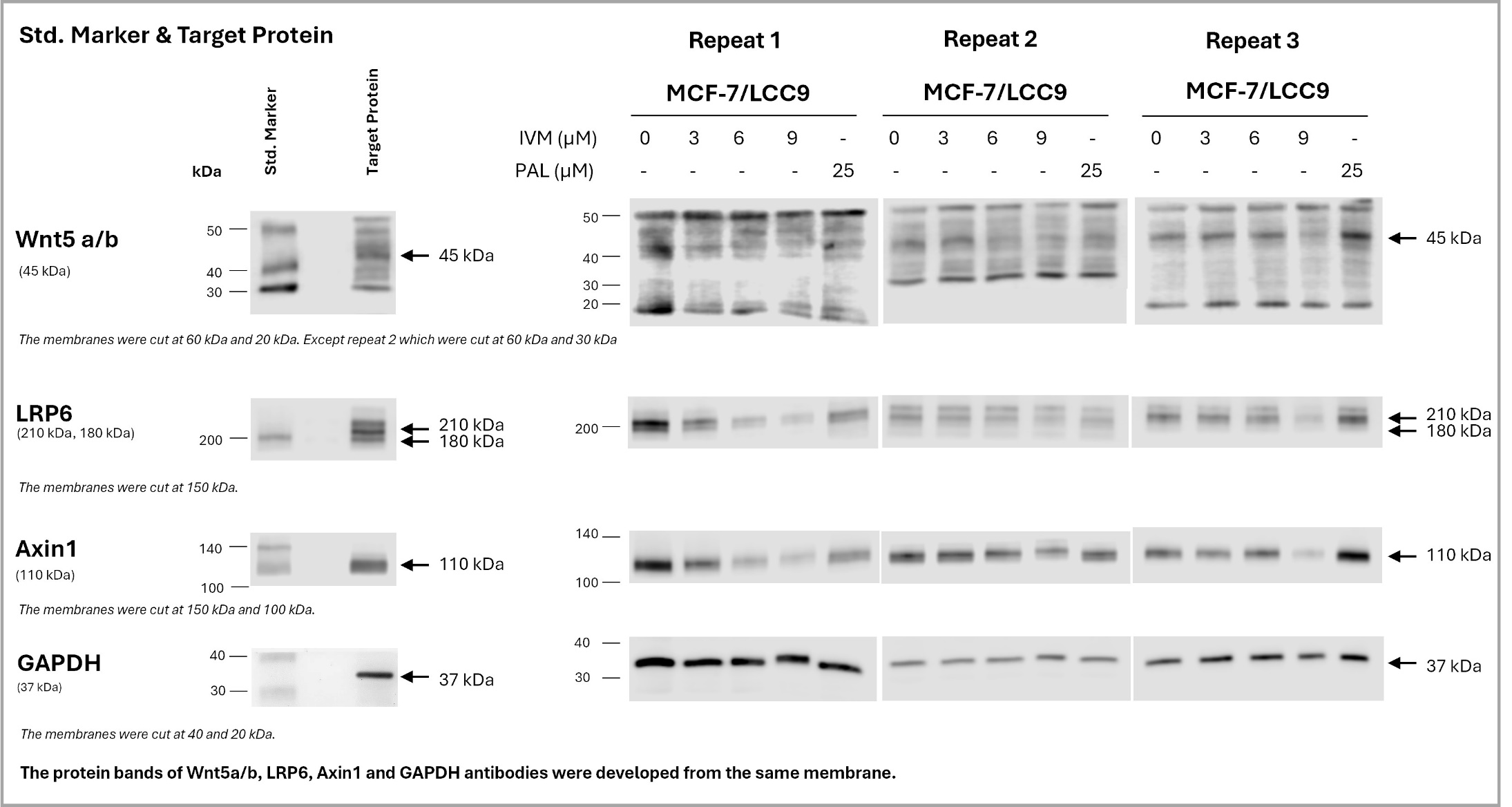


**Figure S2 (B)**: MCF-7/LCC2

**
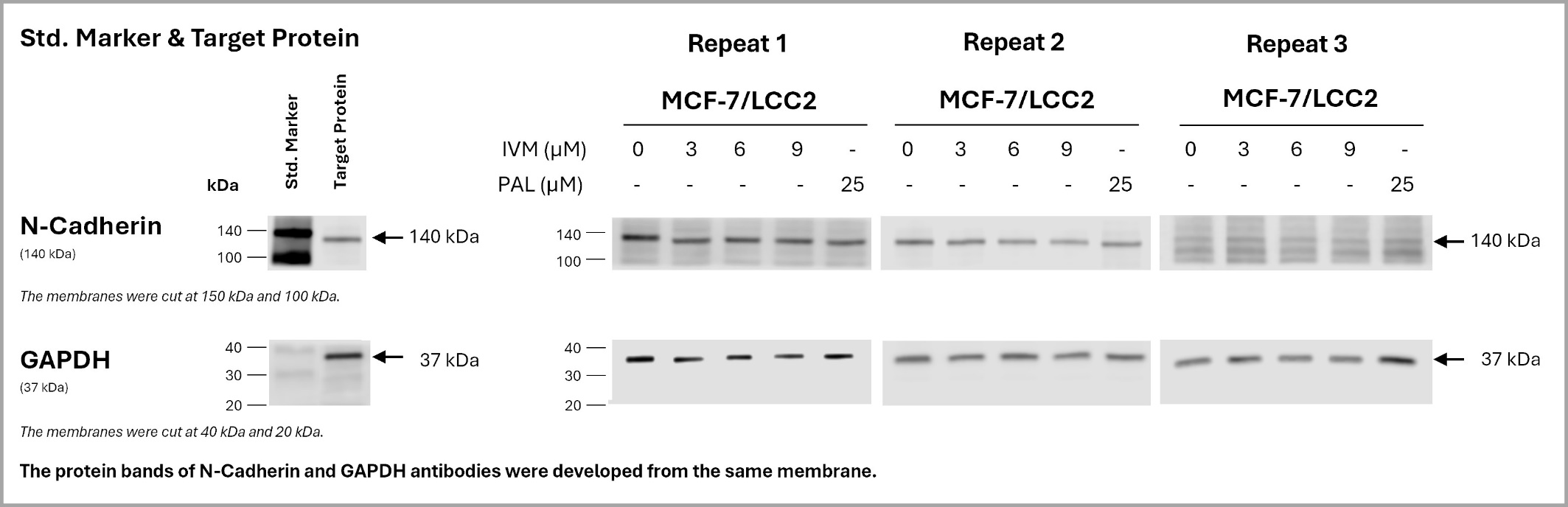
**

**Figure S2 (B)**: MCF-7/LCC9


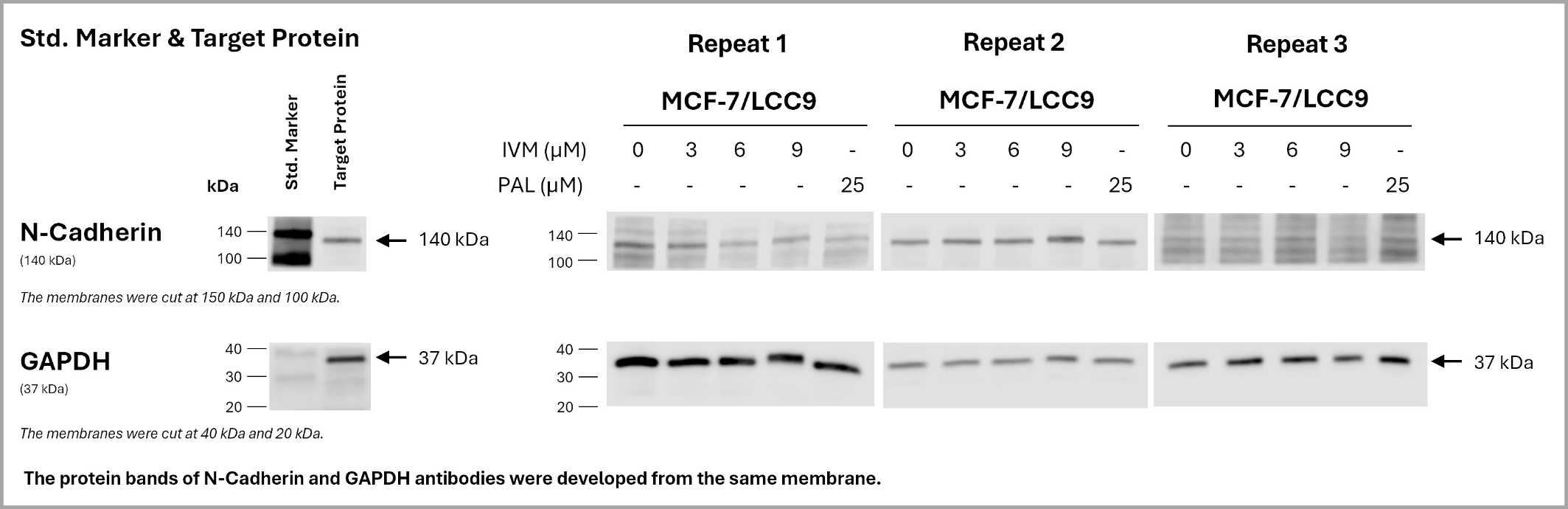


**Figure S2 (C)**: MCF-7/LCC2


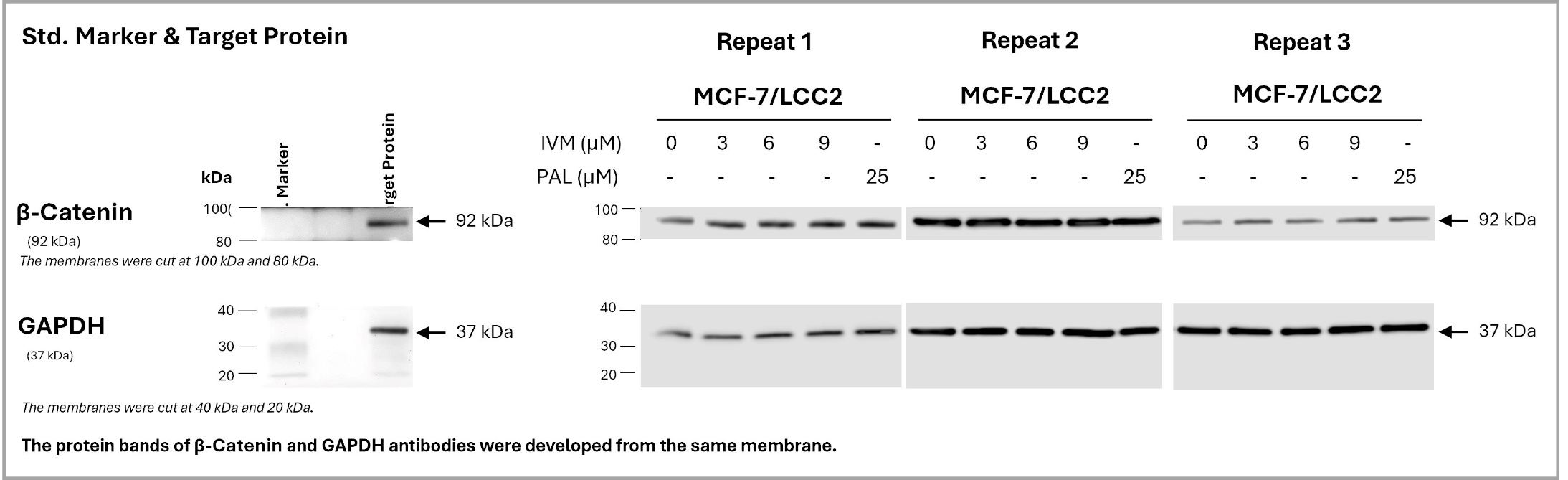


**Figure S2 (C)**: MCF-7/LCC9


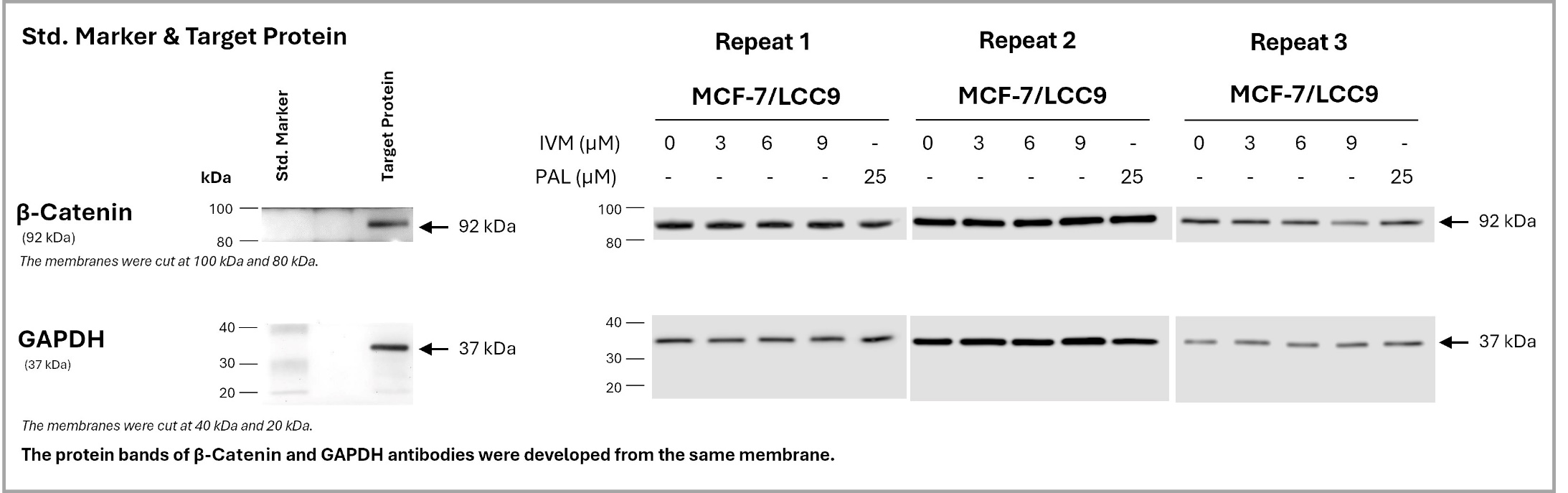


**Figure S2 (D, F)**: MCF-7/LCC2


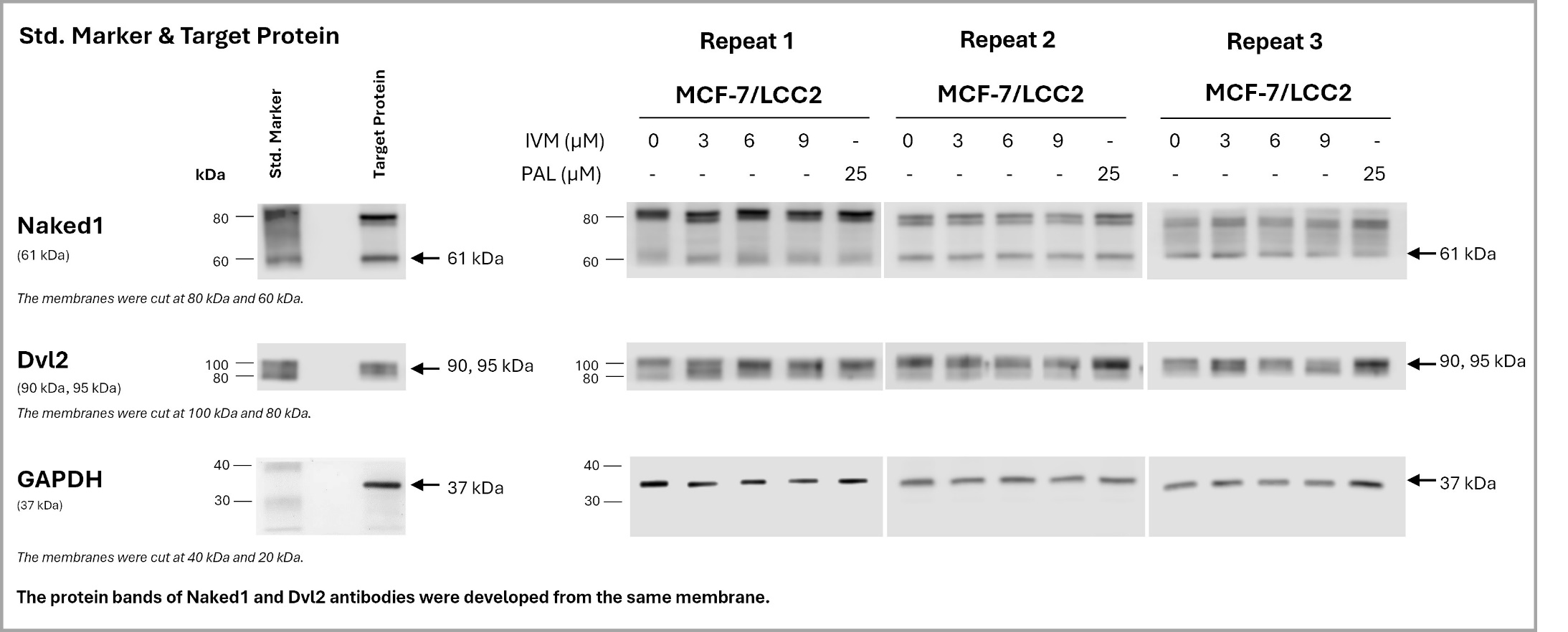


**Figure S2 (D, F)**: MCF-7/LCC9


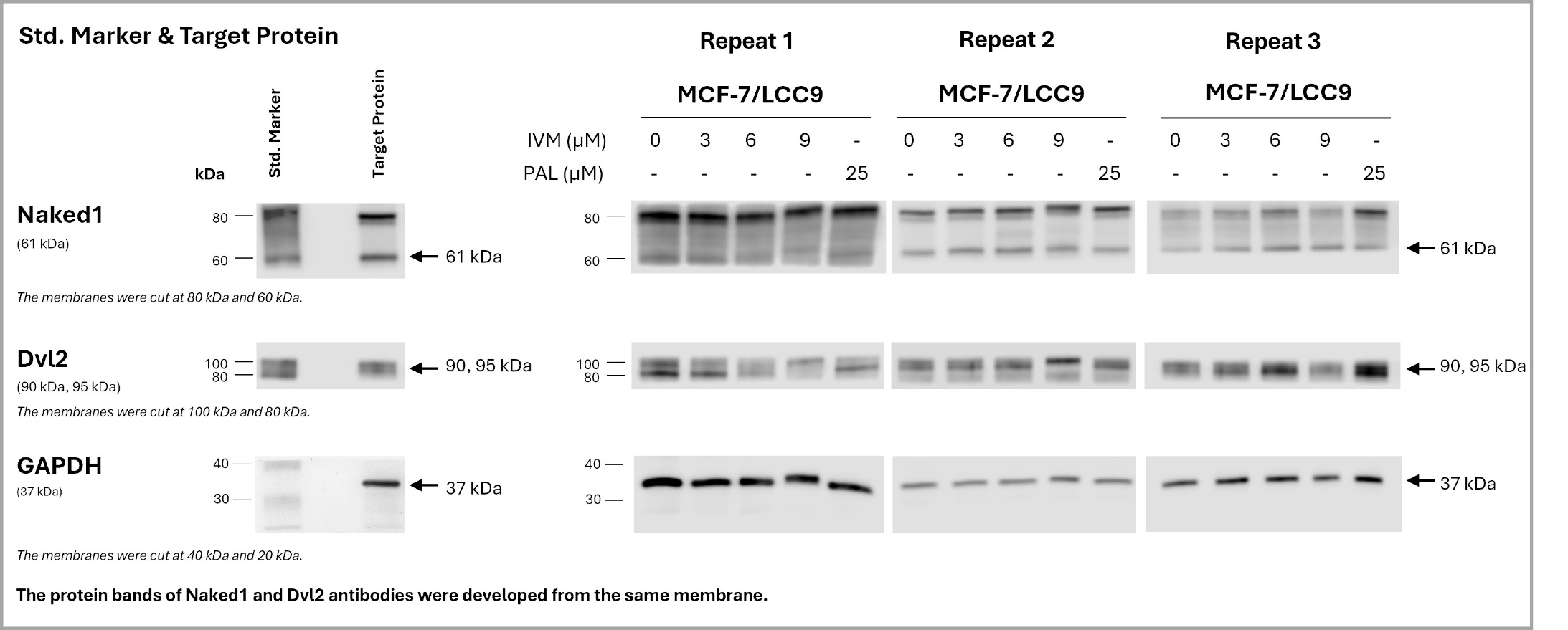


**Figure S2 (E, G):** MCF-7/LCC2


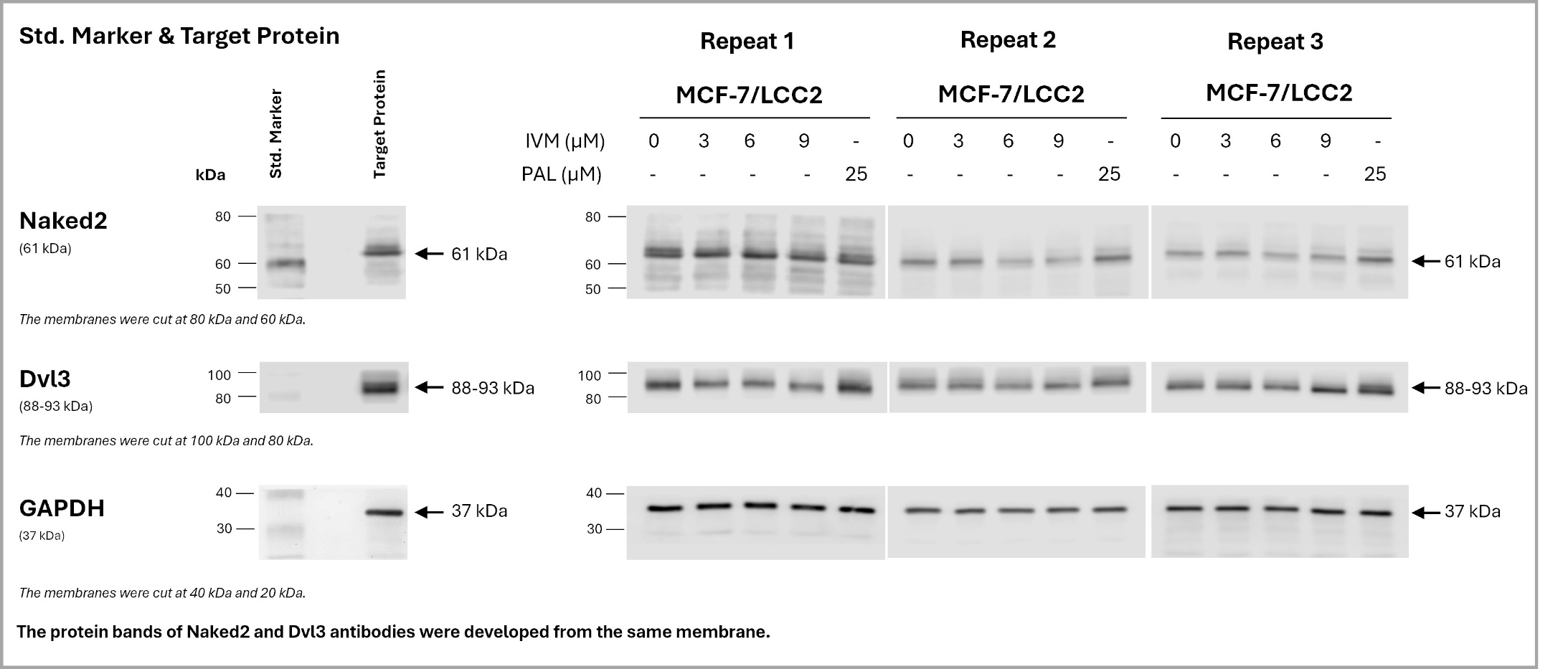


**Figure S2 (E, G)**: MCF-7/LCC9


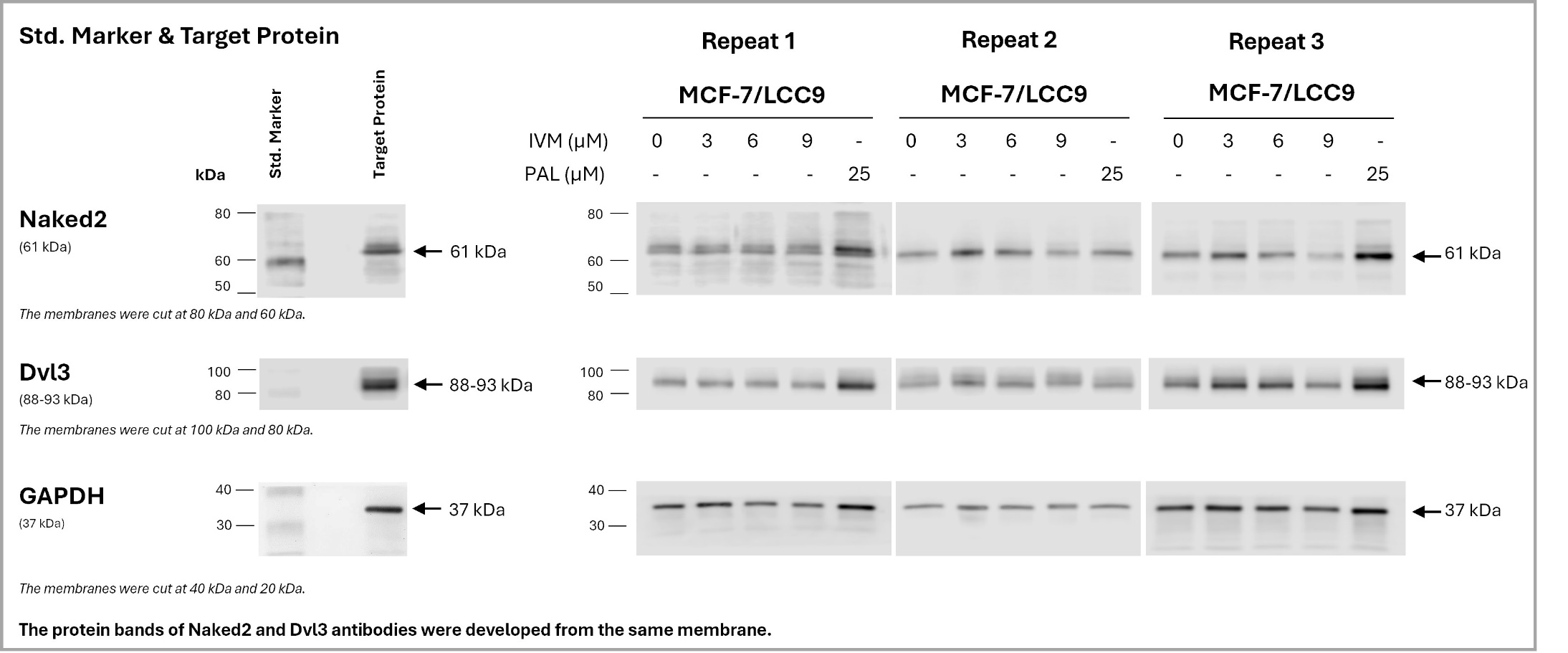


**Figure S3 (B)**: MCF-7


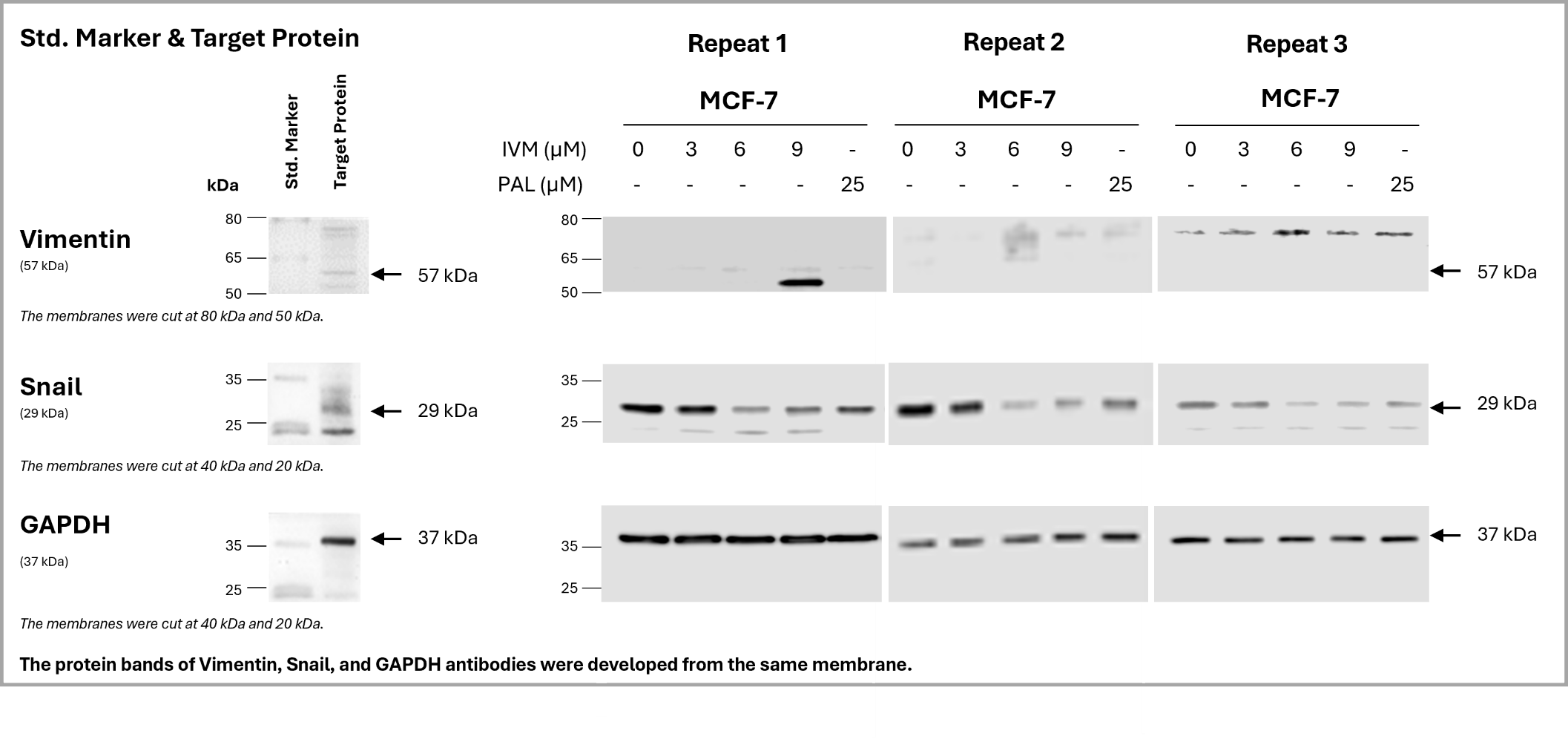


**Figure S4 (B)**: MCF-7


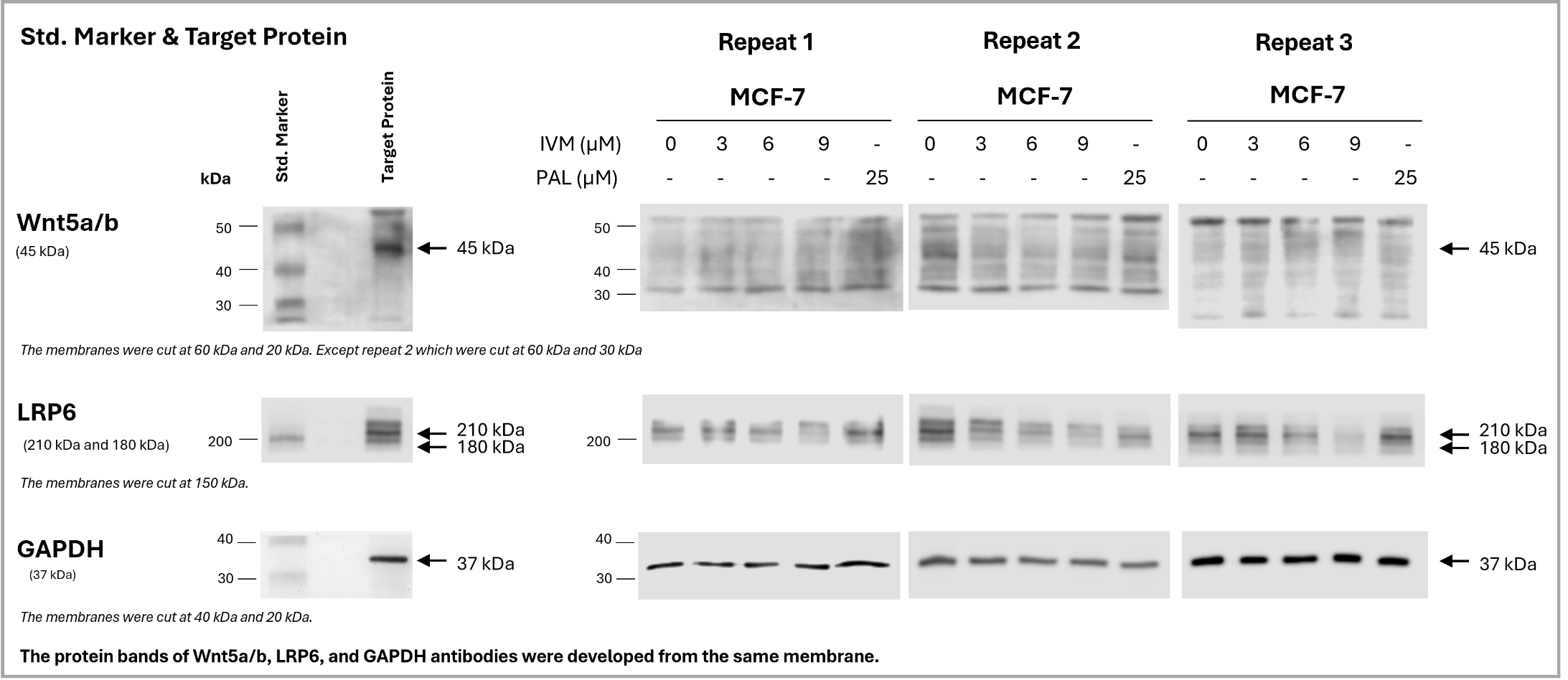


**Figure S4 (C)**: MCF-7


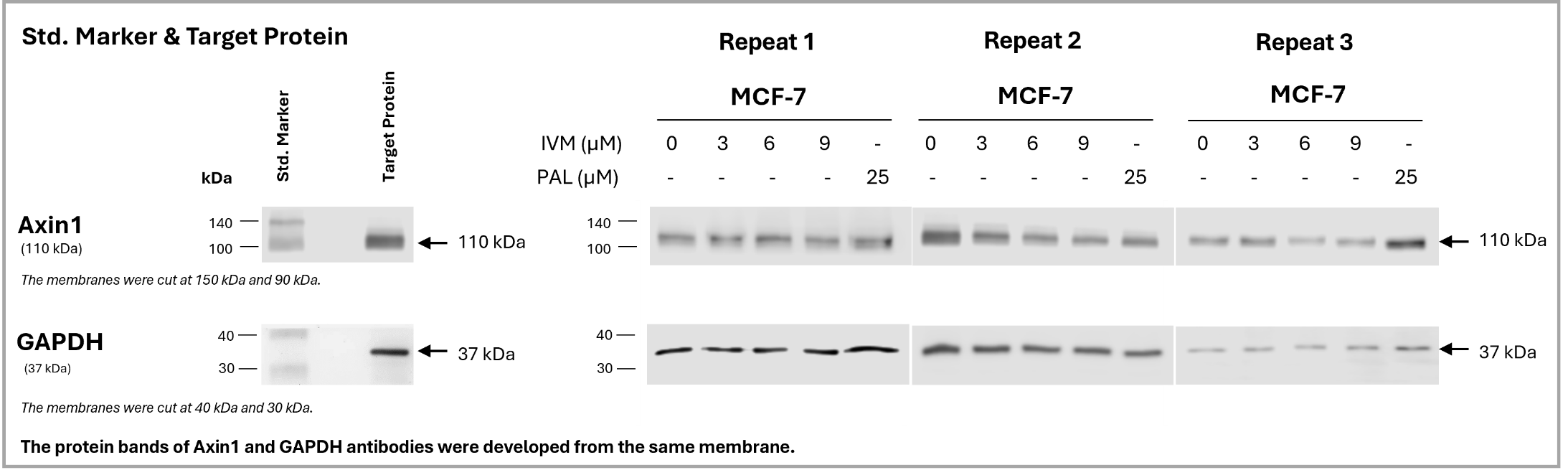


**Figure S5 (B)**: MCF-7


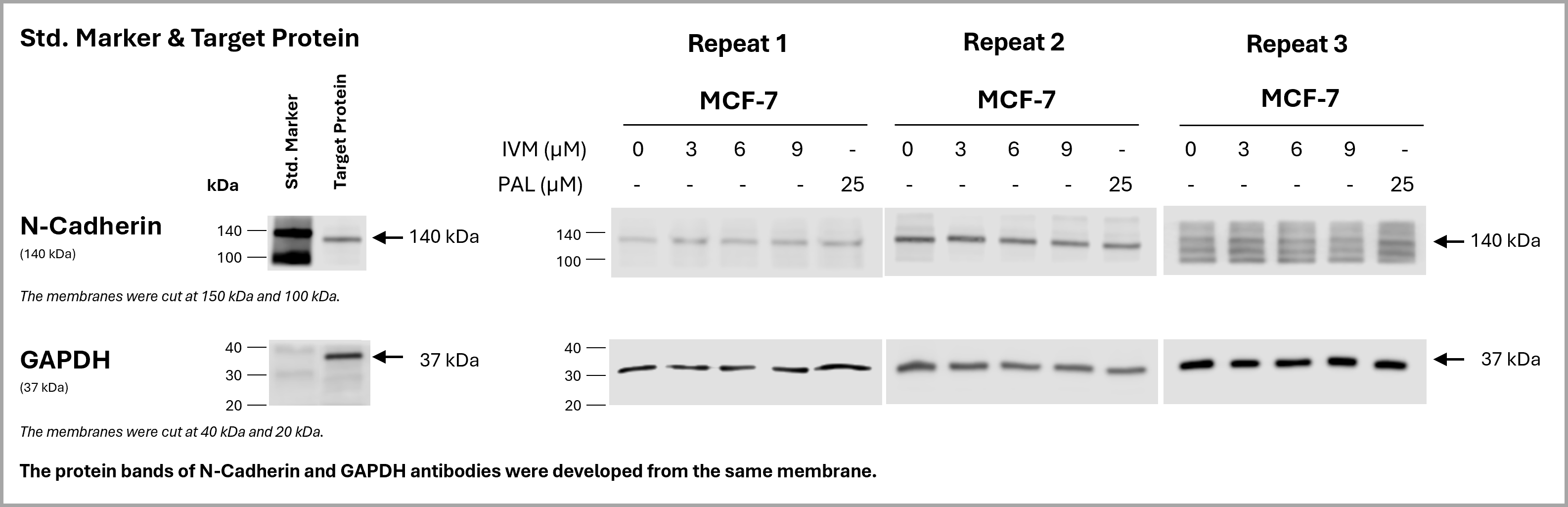


**Figure S5 (C)**: MCF-7


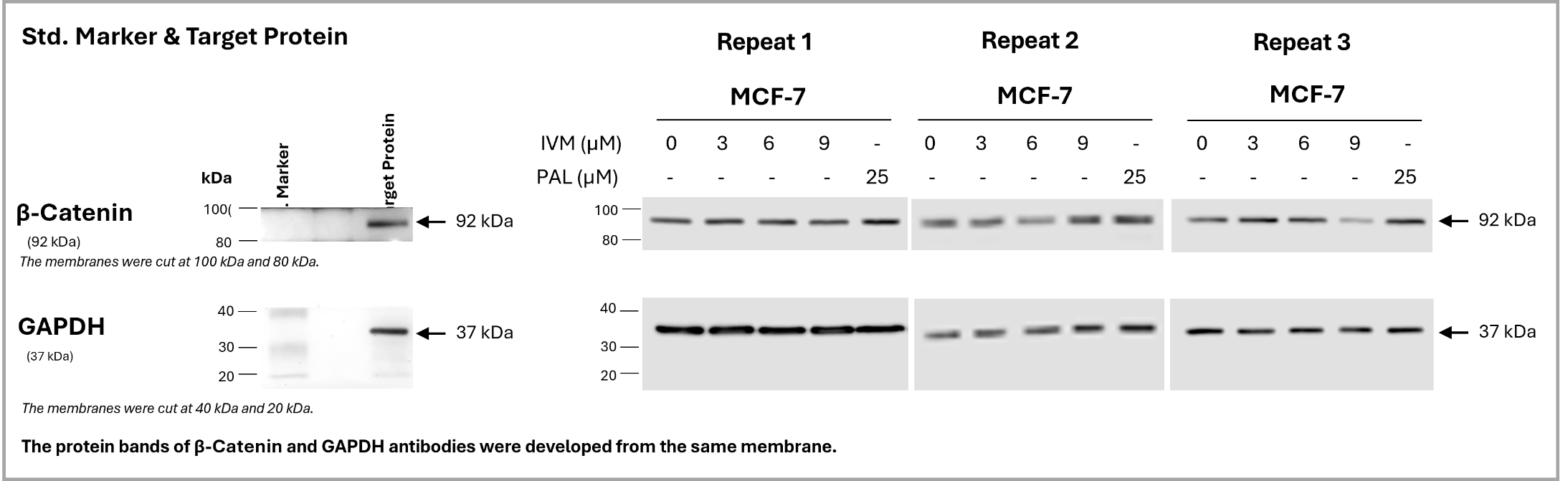


**Figure S5 (D, F)**: MCF-7


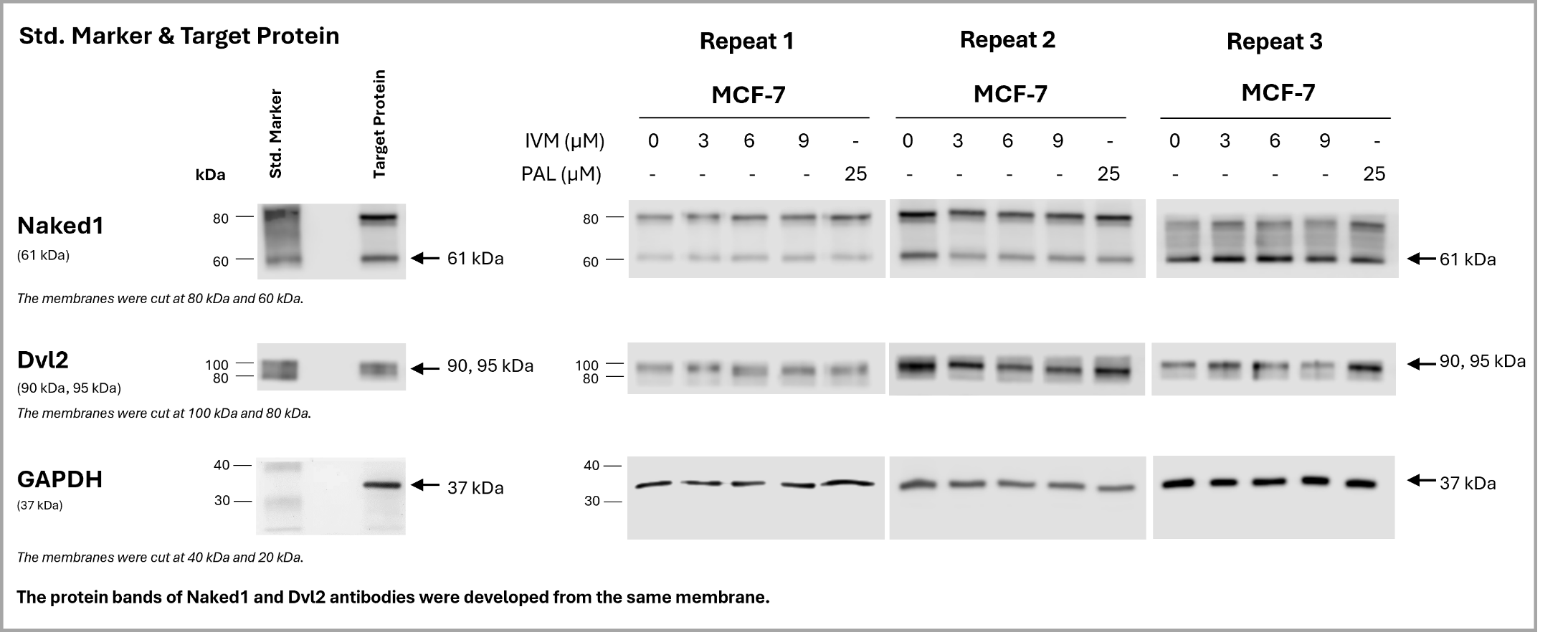


**Figure S5 (E, G)**: MCF-7


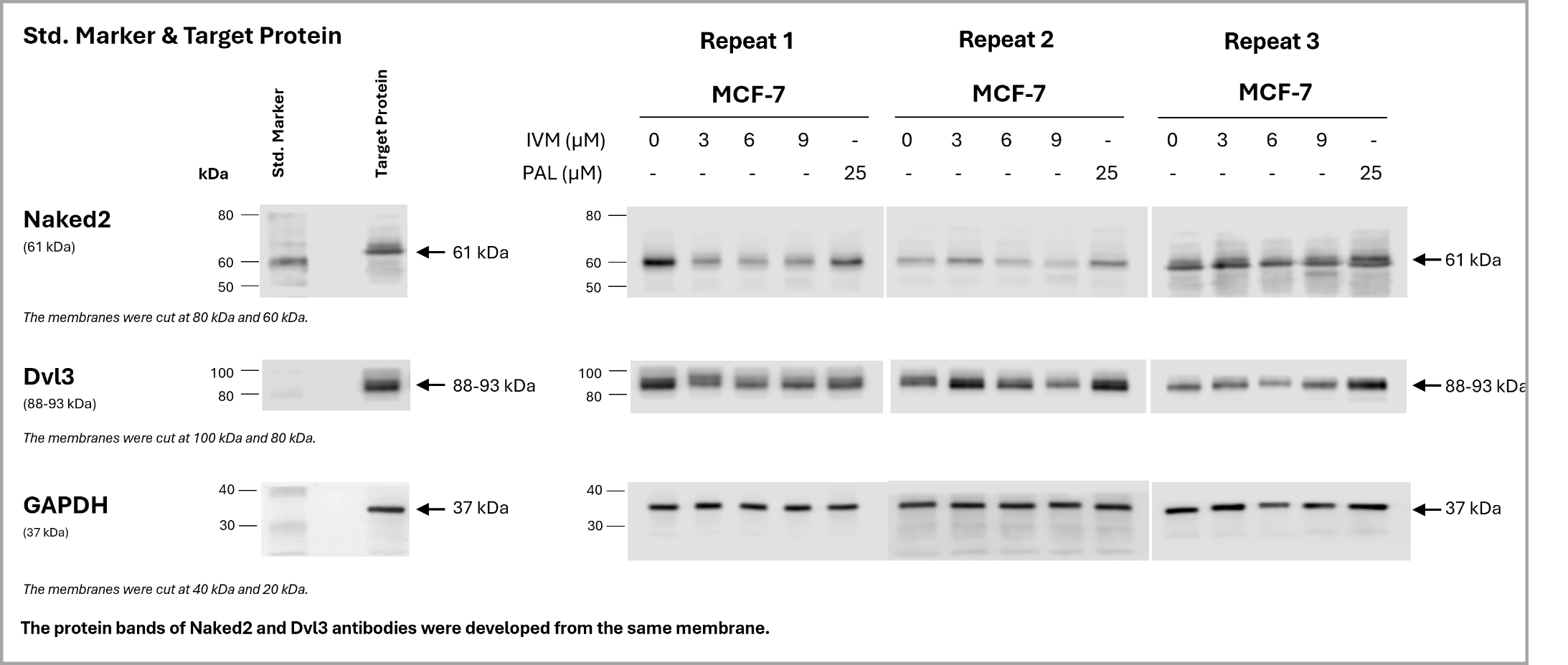

Supplement: S1 File — (DOCX) [file pone.0326742.s009.docx]
