# Supplementary material for: Ivermectin inhibits epithelial-to-mesenchymal transition via Wnt signaling in endocrine-resistant breast cancer cells
Source: PLoS One. 2025 Jun 26;20(6):e0326742. doi: 10.1371/journal.pone.0326742 (PMC12200854; doi:10.1371/journal.pone.0326742)
Supplement: S2 File — (DOCX) [file pone.0326742.s010.docx]

**Supplementary: Raw data and statistical analysis**

**Raw data of Fig.1**

**The effect of IVM on LCC2 cell viability after treatment at various times**

| **IVM conc.  (µM)** | **%Cell Viability  (in relative to non-treatment)** | | |
| --- | --- | --- | --- |
|  | **N1** | **N2** | **N3** |
| 0 | 100.00 | 100.00 | 100.00 |
| 3.125 | 67.77 | 81.79 | 72.43 |
| 6.25 | 46.78 | 43.32 | 42.73 |
| 12.5 | 4.10 | 3.51 | 4.85 |
| 25 | 3.65 | 3.33 | 4.76 |
| 50 | 3.56 | 3.35 | 4.79 |

24 hours after treatment 48 hours after treatment 72 hours after treatment

| **IVM conc.  (µM)** | **%Cell Viability  (in relative to non-treatment)** | | |
| --- | --- | --- | --- |
|  | **N1** | **N2** | **N3** |
| 0 | 100.00 | 100.00 | 100.00 |
| 3.125 | 89.10 | 90.42 | 87.55 |
| 6.25 | 69.47 | 70.17 | 62.96 |
| 12.5 | 35.58 | 41.63 | 37.17 |
| 25 | 12.64 | 14.88 | 14.66 |
| 50 | 9.03 | 8.02 | 11.25 |

| **IVM conc.  (µM)** | **%Cell Viability  (in relative to non-treatment)** | | |
| --- | --- | --- | --- |
|  | **N1** | **N2** | **N3** |
| 0 | 100.00 | 100.00 | 100.00 |
| 3.125 | 80.35 | 84.88 | 69.47 |
| 6.25 | 53.93 | 52.1 | 52.54 |
| 12.5 | 11.73 | 13.32 | 10.96 |
| 25 | 5.36 | 4.75 | 7.53 |
| 50 | 5.33 | 4.56 | 7.03 |

**The effect of IVM on LCC9 cell viability after treatment at various times**

| **IVM conc.  (µM)** | **%Cell Viability  (in relative to non-treatment)** | | |
| --- | --- | --- | --- |
|  | **N1** | **N2** | **N3** |
| 0 | 100.00 | 100.00 | 100.00 |
| 3.125 | 81.42 | 58.98 | 72.73 |
| 6.25 | 58.09 | 49.81 | 45.04 |
| 12.5 | 8.15 | 10.07 | 7.77 |
| 25 | 5.54 | 5.91 | 6.89 |
| 50 | 5.45 | 5.66 | 6.55 |

| **IVM conc.  (µM)** | **%Cell Viability  (in relative to non-treatment)** | | |
| --- | --- | --- | --- |
|  | **N1** | **N2** | **N3** |
| 0 | 100.00 | 100.00 | 100.00 |
| 3.125 | 72.74 | 73.79 | 83.92 |
| 6.25 | 45.68 | 41.8 | 30.66 |
| 12.5 | 5.3 | 5.16 | 5.03 |
| 25 | 4.99 | 5.14 | 4.72 |
| 50 | 4.99 | 4.76 | 4.92 |

24 hours after treatment 48 hours after treatment 72 hours after treatment

| **IVM conc.  (µM)** | **%Cell Viability  (in relative to non-treatment)** | | |
| --- | --- | --- | --- |
|  | **N1** | **N2** | **N3** |
| 0 | 100.00 | 100.00 | 100.00 |
| 3.125 | 88.98 | 75.51 | 81.61 |
| 6.25 | 70.61 | 60.39 | 59.78 |
| 12.5 | 39.74 | 39.66 | 31.45 |
| 25 | 7.83 | 8.83 | 11.59 |
| 50 | 7.19 | 8.24 | 12.14 |

**The effect of IVM on MCF-7 cell viability after treatment at various times**

| **IVM conc.  (µM)** | **%Cell Viability  (in relative to non-treatment)** | | |
| --- | --- | --- | --- |
|  | **N1** | **N2** | **N3** |
| 0 | 100.00 | 100.00 | 100.00 |
| 3.125 | 77.85 | 82.52 | 80.31 |
| 6.25 | 46.25 | 51.97 | 43.59 |
| 12.5 | 10.61 | 13.8 | 10.02 |
| 25 | 7.77 | 7.85 | 5.81 |
| 50 | 7.81 | 7.68 | 5.74 |

| **IVM conc.  (µM)** | **%Cell Viability  (in relative to non-treatment)** | | |
| --- | --- | --- | --- |
|  | **N1** | **N2** | **N3** |
| 0 | 100.00 | 100.00 | 100.00 |
| 3.125 | 75.82 | 69.32 | 71.35 |
| 6.25 | 36.84 | 31.75 | 33.73 |
| 12.5 | 5.59 | 5.27 | 4.51 |
| 25 | 5.25 | 4.94 | 3.73 |
| 50 | 5.25 | 4.94 | 3.75 |

24 hours after treatment 48 hours after treatment 72 hours after treatment

| **IVM conc.  (µM)** | **%Cell Viability  (in relative to non-treatment)** | | |
| --- | --- | --- | --- |
|  | **N1** | **N2** | **N3** |
| 0 | 100.00 | 100.00 | 100.00 |
| 3.125 | 103.42 | 105.64 | 98.64 |
| 6.25 | 82.24 | 82.64 | 75.43 |
| 12.5 | 30.69 | 41.77 | 35.43 |
| 25 | 16.51 | 18.32 | 14.07 |
| 50 | 16.43 | 14.24 | 10.65 |

**Raw data of Fig.2**

**The effect of IVM on the migration of LCC2 after 24 and 48 hours of treatment**

| **Time** | **% Wound healing area in relative to area at 0 hour of treatment** | | | | | | | | | | | | | | | |
| --- | --- | --- | --- | --- | --- | --- | --- | --- | --- | --- | --- | --- | --- | --- | --- | --- |
|  | **IVM 0 µM** | | | **IVM 3 µM** | | | **IVM 6 µM** | | | **IVM 9 µM** | | | **Palbociclib 25 µM** | | | |
|  | **N1** | **N2** | **N3** | **N1** | **N2** | **N3** | **N1** | **N2** | **N3** | **N1** | **N2** | **N3** | **N1** | **N2** | **N3** |  |
| 24 hours | 15.34 | 9.27 | 11.20 | 11.21 | 10.59 | 8.52 | 10.71 | 5.88 | 8.85 | 2.32 | 3.00 | 5.47 | 1.65 | 4.26 | 5.53 |  |
| 48 hours | 22.73 | 12.27 | 19.84 | 16.13 | 12.72 | 13.33 | 11.91 | 4.51 | 9.60 | 3.48 | 0.33 | 3.16 | 5.57 | 5.67 | 7.84 |  |
| *Each "N" represented average counting from 10 areas for comparison at 0, 24, and 48 hours of IVM treatment* | | | | | | | | | | | | | | | | |

**The effect of IVM on the migration of LCC9 after 24 and 48 hours of treatment**

| **Time** | **% Wound healing area in relative to area at 0 hour of treatment** | | | | | | | | | | | | | | |
| --- | --- | --- | --- | --- | --- | --- | --- | --- | --- | --- | --- | --- | --- | --- | --- |
|  | **IVM 0 µM** | | | **IVM 3 µM** | | | **IVM 6 µM** | | | **IVM 9 µM** | | | **Palbociclib 25 µM** | | |
|  | **N1** | **N2** | **N3** | **N1** | **N2** | **N3** | **N1** | **N2** | **N3** | **N1** | **N2** | **N3** | **N1** | **N2** | **N3** |
| 24 hours | 16.63 | 16.12 | 17.48 | 14.50 | 14.37 | 14.85 | 12.47 | 11.24 | 15.08 | 3.35 | 12.31 | 8.58 | 1.56 | 8.51 | 4.19 |
| 48 hours | 22.46 | 19.40 | 20.85 | 18.55 | 17.45 | 16.26 | 16.22 | 13.41 | 16.43 | 4.57 | 11.09 | 6.74 | 4.10 | 13.56 | 10.93 |
| *Each "N" represented average counting from 10 areas for comparison at 0, 24, and 48 hours of IVM treatment* | | | | | | | | | | | | | | | |

**Raw data of Fig.3**

**The effect of IVM on the invasion of breast cancer cells after 24 hours of treatment**

| **Cell** | **% Cell invasion in relative to non-treatment** | | | | | | | | | | | | | | |
| --- | --- | --- | --- | --- | --- | --- | --- | --- | --- | --- | --- | --- | --- | --- | --- |
|  | **IVM 0 µM** | | | **IVM 3 µM** | | | **IVM 6 µM** | | | **IVM 9 µM** | | | **Palbociclib 25 µM** | | |
|  | **N1** | **N2** | **N3** | **N1** | **N2** | **N3** | **N1** | **N2** | **N3** | **N1** | **N2** | **N3** | **N1** | **N2** | **N3** |
| **LCC2** | 100.00 | 100.00 | 100.00 | 103.00 | 50.00 | 80.00 | 21.00 | 34.00 | 91.00 | 25.00 | 35.00 | 53.00 | 46.00 | 50.00 | 80.00 |
| **LCC9** | 100.00 | 100.00 | 100.00 | 102.00 | 77.00 | 105.00 | 87.00 | 109.00 | 91.00 | 58.00 | 53.00 | 84.00 | 51.00 | 76.00 | 79.00 |
| *Each "N" represented the average of counting from 10 fields of each treated group for 24 hours after treatment* | | | | | | | | | | | | | | | |

**Supplementary data Fig.S1: %Cell viability after the combination treatment between 4-OHT and IVM for 72 hours**

**The combined treatment effect between 4-OHT and IVM in LCC2 cells in relative to non-treatment**

| **4-OHT Conc. (µM)** | **% Cell viability** (Mean ± SEM) | | | |
| --- | --- | --- | --- | --- |
|  | **IVM 0 µM** | **IVM 4 µM** | **IVM 7 µM** | **IVM 9 µM** |
| 0 | 100 ± 0 | 52.53 ± 3.95 | 34.42 ± 2.88 | 24.46 ± 1.38 |
| 2.5 | 81.77 ± 4.12 | 47.12 ± 3.17 | 31.91 ± 1.98 | 26.48 ± 1.50 |
| 5 | 79.58 ± 3.92 | 34.34 ± 5.34 | 28.59 ± 1.88 | 22.00 ± 0.92 |
| 7.5 | 71.84 ± 4.55 | 20.68 ± 2.06 | 7.58 ± 1.73 | 3.40 ± 0.91 |
| 10 | 32.13 ± 6.29 | 16.59 ± 4.20 | 1.60 ± 0.12 | 1.22 ± 0.09 |

**The combined treatment effect between 4-OHT and IVM in LCC9 cells in relative to non-treatment**

| **4-OHT Conc. (µM)** | **% Cell viability** (Mean ± SEM) | | | |
| --- | --- | --- | --- | --- |
|  | **IVM 0 µM** | **IVM 3 µM** | **IVM 5 µM** | **IVM 7 µM** |
| 0 | 100 ± 0 | 63.83 ± 2.23 | 37.77 ± 0.88 | 22.16 ± 1.15 |
| 2.5 | 74.36 ± 6.97 | 42.03 ± 3.24 | 27.9 ± 3.11 | 21.51 ± 0.34 |
| 5 | 71.89 ± 2.24 | 41.38 ± 1.02 | 18.2 ± 4.64 | 12.88 ± 1.01 |
| 7.5 | 62.35 ± 2.42 | 29.02 ± 2.06 | 6.77 ± 1.17 | 3.20 ± 0.57 |
| 10 | 23.65 ± 1.54 | 7.00 ± 2.06 | 2.24 ± 0.01 | 1.35 ± 0.14 |

**The combined treatment effect between 4-OHT and IVM in MCF-7 cells in relative to non-treatment**

| **4-OHT Conc. (µM)** | **% Cell viability** (Mean ± SEM) | | | |
| --- | --- | --- | --- | --- |
|  | **IVM 0 µM** | **IVM 0.5 µM** | **IVM 2 µM** | **IVM 8 µM** |
| 0 | 100± 0 | 86.3 ± 10.03 | 97.98 ± 6.24 | 37 ± 3.32 |
| 2.5 | 69.11 ± 0.9 | 72.34 ± 5.36 | 82.92 ± 0.26 | 36.3 ± 2.91 |
| 5 | 64.84 ± 3.3 | 64.55 ± 6.83 | 58.18 ± 1.95 | 15.8 ± 2.36 |
| 7.5 | 54.47 ± 1.42 | 45.6 ± 7.85 | 42.4 ± 0.22 | 6.18 ± 1.43 |
| 10 | 29.90 ± 0.40 | 17.1 ± 6.19 | 17.13 ± 1.33 | 1.52 ± 0.27 |

**Supplementary data: Quantitative protein expression analysis by western blot in all figures**

**The effect of IVM in LCC2 cells after 24 hours of treatment in relative to non-treatment**

| **Target protein** | **Protein expression in relative to non-treatment (Mean ± SEM)** | | | | |
| --- | --- | --- | --- | --- | --- |
|  | **IVM 0 µM** | **IVM 3 µM** | **IVM 6 µM** | **IVM 9 µM** | **Palbociclib 25 µM** |
| Vimenatin | 1.00 ± 0.00 | 0.97 ± 0.13 | 0.23 ± 0.06 | 0.08 ± 0.03 | 0.91 ± 0.25 |
| Snail | 1.00 ± 0.00 | 0.51 ± 0.09 | 0.29 ± 0.09 | 0.21 ± 0.08 | 0.62 ± 0.08 |
| Wnt5a/b | 1.00 ± 0.00 | 1.02 ± 0.28 | 0.59 ± 0.2 | 0.39 ± 0.08 | 0.69 ± 0.16 |
| LRP6 | 1.00 ± 0.00 | 0.68 ± 0.04 | 0.46 ± 0.05 | 0.35 ± 0.09 | 1.32 ± 0.23 |
| Axin1 | 1.00 ± 0.00 | 0.84 ± 0.22 | 0.48 ± 0.13 | 0.57 ± 0.1 | 0.91 ± 0.27 |
| N-Cadherin | 1.00 ± 0.00 | 0.9 ± 0.08 | 1.07 ± 0.27 | 0.84 ± 0.02 | 0.88 ± 0.08 |
| β-Catenin | 1.00 ± 0.00 | 1.02 ± 0.19 | 0.93 ± 0.08 | 0.72 ± 0.25 | 0.89 ± 0.08 |
| Naked1 | 1.00 ± 0.00 | 1.13 ± 0.04 | 0.98 ± 0.06 | 0.96 ± 0.03 | 0.82 ± 0.14 |
| Naked2 | 1.00 ± 0.00 | 1.26 ± 0.05 | 0.9 ± 0.18 | 0.97 ± 0.12 | 1.4 ± 0.2 |
| Dvl2 | 1.00 ± 0.00 | 1.18 ± 0.06 | 1.35 ± 0.44 | 1.19 ± 0.17 | 1.51 ± 0.22 |
| Dvl3 | 1.00 ± 0.00 | 0.88 ± 0.1 | 0.73 ± 0.06 | 0.97 ± 0.13 | 1.14 ± 0.1 |

**The effect of IVM in LCC9 cells after 24 hours of treatment in relative to non-treatment**

| **Target protein** | **Protein expression in relative to non-treatment (Mean ± SEM)** | | | | |
| --- | --- | --- | --- | --- | --- |
|  | **IVM 0 µM** | **IVM 3 µM** | **IVM 6 µM** | **IVM 9 µM** | **Palbociclib 25 µM** |
| Vimenatin | 1.00 ± 0.00 | 0.26 ± 0.11 | 0.28 ± 0.17 | 0.2 ± 0.21 | 1.21 ± 0.28 |
| Snail | 1.00 ± 0.00 | 0.59 ± 0.06 | 0.47 ± 0.06 | 0.35 ± 0.03 | 0.45 ± 0.02 |
| Wnt5a/b | 1.00 ± 0.00 | 1.15 ± 0.08 | 0.73 ± 0.23 | 0.63 ± 0.12 | 1.06 ± 0.3 |
| LRP6 | 1.00 ± 0.00 | 1.06 ± 0.14 | 0.76 ± 0.11 | 0.51 ± 0.07 | 1.13 ± 0.27 |
| Axin1 | 1.00 ± 0.00 | 0.96 ± 0.19 | 0.7 ± 0.08 | 0.39 ± 0.06 | 1.06 ± 0.17 |
| N-Cadherin | 1.00 ± 0.00 | 1.26 ± 0.29 | 1.03 ± 0.18 | 1.5 ± 0.31 | 0.87 ± 0.1 |
| β-Catenin | 1.00 ± 0.00 | 0.75 ± 0.18 | 0.78 ± 0.09 | 0.63 ± 0.2 | 0.61 ± 0.06 |
| Naked1 | 1.00 ± 0.00 | 1.33 ± 0.14 | 1.45 ± 0.08 | 1.18 ± 0.28 | 1.1 ± 0.04 |
| Naked2 | 1.00 ± 0.00 | 1.17 ± 0.21 | 1.17 ± 0.2 | 0.83 ± 0.2 | 1.31 ± 0.3 |
| Dvl2 | 1.00 ± 0.00 | 1.12 ± 0.25 | 0.97 ± 0.08 | 0.45 ± 0.23 | 1.08 ± 0.1 |
| Dvl3 | 1.00 ± 0.00 | 1.12 ± 0.14 | 1.27 ± 0.04 | 1.17 ± 0.08 | 1.38 ± 0.21 |

**The effect of IVM in MCF-7 cells after 24 hours of treatment in relative to non-treatment**

| **Target protein** | **Protein expression in relative to non-treatment (Mean ± SEM)** | | | | |
| --- | --- | --- | --- | --- | --- |
|  | **IVM 0 µM** | **IVM 3 µM** | **IVM 6 µM** | **IVM 9 µM** | **Palbociclib 25 µM** |
| Snail | 1.00 ± 0.00 | 0.81 ± 0.07 | 0.31 ± 0.06 | 0.39 ± 0.09 | 0.44 ± 0.08 |
| Wnt5a/b | 1.00 ± 0.00 | 1.14 ± 0.32 | 0.84 ± 0.24 | 0.89 ± 0.04 | 0.88 ± 0.21 |
| LRP6 | 1.00 ± 0.00 | 0.97 ± 0.14 | 0.75 ± 0.11 | 0.42 ± 0.2 | 1.29 ± 0.36 |
| Axin1 | 1.00 ± 0.00 | 0.97 ± 0.06 | 0.86 ± 0.25 | 0.74 ± 0.11 | 0.88 ± 0.16 |
| N-Cadherin | 1.00 ± 0.00 | 1.67 ± 0.23 | 1.29 ± 0.08 | 1.12 ± 0.23 | 1.18 ± 0.08 |
| β-Catenin | 1.00 ± 0.00 | 1.29 ± 0.25 | 1.08 ± 0.27 | 0.87 ± 0.16 | 1.18 ± 0.25 |
| Naked1 | 1.00 ± 0.00 | 1.32 ± 0.35 | 1.32 ± 0.28 | 1.07 ± 0.1 | 1.11 ± 0.09 |
| Naked2 | 1.00 ± 0.00 | 0.99 ± 0.38 | 0.88 ± 0.37 | 0.72 ± 0.21 | 1.05 ± 0.32 |
| Dvl2 | 1.00 ± 0.00 | 1.29 ± 0.25 | 1.19 ± 0.22 | 0.87 ± 0.15 | 1.44 ± 0.59 |
| Dvl3 | 1.00 ± 0.00 | 0.88 ± 0.29 | 0.88 ± 0.18 | 0.96 ± 0.15 | 1.43 ± 0.31 |

**Supplementary Table:** Statistical Analysis

| **Figure** | **Assay** | **Experimental detail/ Target Protein** | **Cell lines** | **Group comparisons** | **Mean Diff.** | **95% CI of diff.** | **P value** |
| --- | --- | --- | --- | --- | --- | --- | --- |
| Figure 2B | Migration | Treatment 24 h. | MCF-7/LCC2 | IVM 0 µM vs. IVM 3 µM | 1.8300 | -3.465 to 7.125 | 0.6468 |
|  |  |  |  | IVM 0 µM vs. IVM 6 µM | 3.4570 | -1.838 to 8.752 | 0.2169 |
|  |  |  |  | IVM 0 µM vs. IVM 9 µM | 8.3400 | 3.045 to 13.63 | 0.0049 |
|  |  |  |  | IVM 0 µM vs. PAL 25 µM | -8.1230 | -14.02 to -2.227 | 0.0187 |
|  |  | Treatment 48 h. | MCF-7/LCC2 | IVM 0 µM vs. IVM 3 µM | 4.2200 | -4.078 to 12.52 | 0.3797 |
|  |  |  |  | IVM 0 µM vs. IVM 6 µM | 9.6070 | 1.308 to 17.91 | 0.0257 |
|  |  |  |  | IVM 0 µM vs. IVM 9 µM | 15.9600 | 7.658 to 24.26 | 0.0014 |
|  |  |  |  | IVM 0 µM vs. PAL 25 µM | -11.9200 | -20.82 to -3.020 | 0.0205 |
| Figure 2D | Migration | Treatment 24 h. | MCF-7/LCC9 | IVM 0 µM vs. IVM 3 µM | 2.1700 | -3.665 to 8.005 | 0.5996 |
|  |  |  |  | IVM 0 µM vs. IVM 6 µM | 3.8130 | -2.022 to 9.649 | 0.2163 |
|  |  |  |  | IVM 0 µM vs. IVM 9 µM | 8.6630 | 2.828 to 14.50 | 0.0069 |
|  |  |  |  | IVM 0 µM vs. PAL 25 µM | -11.9900 | -17.72 to -6.258 | 0.0044 |
|  |  | Treatment 48 h. | MCF-7/LCC9 | IVM 0 µM vs. IVM 3 µM | 3.4830 | -1.438 to 8.404 | 0.1730 |
|  |  |  |  | IVM 0 µM vs. IVM 6 µM | 5.5500 | 0.6289 to 10.47 | 0.0291 |
|  |  |  |  | IVM 0 µM vs. IVM 9 µM | 13.4400 | 8.516 to 18.36 | 0.0001 |
|  |  |  |  | IVM 0 µM vs. PAL 25 µM | -11.3700 | -19.58 to -3.171 | 0.0183 |
| Figure 3B | Invasion | Treatment 24 h. | MCF-7/LCC2 | IVM 0 µM vs. IVM 3 µM | 22.3300 | -33.98 to 78.64 | 0.5560 |
|  |  |  |  | IVM 0 µM vs. IVM 6 µM | 51.3300 | -4.975 to 107.6 | 0.0729 |
|  |  |  |  | IVM 0 µM vs. IVM 9 µM | 62.3300 | 6.025 to 118.6 | 0.0318 |
|  |  |  |  | IVM 0 µM vs. PAL 25 µM | -41.6700 | -70.54 to -12.80 | 0.0160 |
| Figure 3D | Invasion | Treatment 24 h. | MCF-7/LCC9 | IVM 0 µM vs. IVM 3 µM | 5.3330 | -24.66 to 35.32 | 0.9179 |
|  |  |  |  | IVM 0 µM vs. IVM 6 µM | 4.3330 | -25.66 to 34.32 | 0.9523 |
|  |  |  |  | IVM 0 µM vs. IVM 9 µM | 35.0000 | 5.011 to 64.99 | 0.0247 |
|  |  |  |  | IVM 0 µM vs. PAL 25 µM | -31.3300 | -55.98 to -6.690 | 0.0242 |

| **Figure** | **Assay** | **Experimental detail/ Target Protein** | **Cell lines** | **Group comparisons** | **Mean Diff.** | **95% CI of diff.** | **P value** |
| --- | --- | --- | --- | --- | --- | --- | --- |
| Figure 4B | Western Blot | N-Cadherin | MCF-7/LCC2 | IVM 0 µM vs. IVM 3 µM | 0.0967 | -0.4796 to 0.6729 | 0.9294 |
|  |  |  |  | IVM 0 µM vs. IVM 6 µM | -0.0700 | -0.6463 to 0.5063 | 0.9703 |
|  |  |  |  | IVM 0 µM vs. IVM 9 µM | 0.1567 | -0.4196 to 0.7329 | 0.7783 |
|  |  |  |  | IVM 0 µM vs. PAL 25 µM | -0.1167 | -0.3301 to 0.09680 | 0.2038 |
|  |  |  | MCF-7/LCC9 | IVM 0 µM vs. IVM 3 µM | -0.2567 | -1.191 to 0.6781 | 0.7737 |
|  |  |  |  | IVM 0 µM vs. IVM 6 µM | -0.0267 | -0.9614 to 0.9081 | 0.9996 |
|  |  |  |  | IVM 0 µM vs. IVM 9 µM | -0.5000 | -1.4350 to 0.4347 | 0.3443 |
|  |  |  |  | IVM 0 µM vs. PAL 25 µM | -0.1300 | -0.3939 to 0.1339 | 0.2432 |
| Figure 4C | Western Blot | Vimentin | MCF-7/LCC2 | IVM 0 µM vs. IVM 3 µM | 0.0333 | -0.2685 to 0.3351 | 0.9772 |
|  |  |  |  | IVM 0 µM vs. IVM 6 µM | 0.7733 | 0.4715 to 1.0750 | 0.0002 |
|  |  |  |  | IVM 0 µM vs. IVM 9 µM | 0.9233 | 0.6215 to 1.2250 | <0.0001 |
|  |  |  |  | IVM 0 µM vs. PAL 25 µM | -0.0867 | -0.7917 to 0.6183 | 0.7500 |
|  |  |  | MCF-7/LCC9 | IVM 0 µM vs. IVM 3 µM | 0.7433 | 0.1525 to 1.3340 | 0.0170 |
|  |  |  |  | IVM 0 µM vs. IVM 6 µM | 0.7200 | 0.1292 to 1.3110 | 0.0200 |
|  |  |  |  | IVM 0 µM vs. IVM 9 µM | 0.8033 | 0.2125 to 1.3940 | 0.0113 |
|  |  |  |  | IVM 0 µM vs. PAL 25 µM | 0.2133 | -0.5531 to 0.9798 | 0.4828 |
| Figure 4D | Western Blot | Snail | MCF-7/LCC2 | IVM 0 µM vs. IVM 3 µM | 0.4933 | 0.1810 to 0.8057 | 0.0048 |
|  |  |  |  | IVM 0 µM vs. IVM 6 µM | 0.7133 | 0.4010 to 1.0260 | 0.0005 |
|  |  |  |  | IVM 0 µM vs. IVM 9 µM | 0.7867 | 0.4743 to 1.0990 | 0.0002 |
|  |  |  |  | IVM 0 µM vs. PAL 25 µM | -0.3767 | -0.5931 to -0.1602 | 0.0085 |
|  |  |  | MCF-7/LCC9 | IVM 0 µM vs. IVM 3 µM | 0.4133 | 0.2399 to 0.5868 | 0.0003 |
|  |  |  |  | IVM 0 µM vs. IVM 6 µM | 0.5300 | 0.3566 to 0.7034 | <0.0001 |
|  |  |  |  | IVM 0 µM vs. IVM 9 µM | 0.6533 | 0.4799 to 0.8268 | <0.0001 |
|  |  |  |  | IVM 0 µM vs. PAL 25 µM | -0.5467 | -0.6030 to -0.4904 | <0.0001 |

| **Figure** | **Assay** | **Experimental detail/ Target Protein** | **Cell lines** | **Group comparisons** | **Mean Diff.** | **95% CI of diff.** | **P value** |
| --- | --- | --- | --- | --- | --- | --- | --- |
| Figure 5B | Western Blot | Wnt5a/b | MCF-7/LCC2 | IVM 0 µM vs. IVM 3 µM | 0.0200 | -0.7655 to 0.8055 | 0.9470 |
|  |  |  |  | IVM 0 µM vs. IVM 6 µM | -0.4100 | -0.9632 to 0.1432 | 0.1087 |
|  |  |  |  | IVM 0 µM vs. IVM 9 µM | -0.6067 | -0.8195 to -0.3938 | 0.0014 |
|  |  |  |  | IVM 0 µM vs. PAL 25 µM | -0.3067 | -0.7405 to 0.1271 | 0.1212 |
|  |  |  | MCF-7/LCC9 | IVM 0 µM vs. IVM 3 µM | 0.1533 | -0.0736 to 0.3802 | 0.1338 |
|  |  |  |  | IVM 0 µM vs. IVM 6 µM | -0.2733 | -0.9214 to 0.3747 | 0.3066 |
|  |  |  |  | IVM 0 µM vs. IVM 9 µM | -0.3700 | -0.6894 to -0.0506 | 0.0324 |
|  |  |  |  | IVM 0 µM vs. PAL 25 µM | 0.0600 | -0.7827 to 0.9027 | 0.8529 |
| Figure 5C | Western Blot | LRP6 | MCF-7/LCC2 | IVM 0 µM vs. IVM 3 µM | 0.3233 | 0.0939 to 0.5528 | 0.0093 |
|  |  |  |  | IVM 0 µM vs. IVM 6 µM | 0.5433 | 0.3139 to 0.7728 | 0.0004 |
|  |  |  |  | IVM 0 µM vs. IVM 9 µM | 0.6467 | 0.4172 to 0.8761 | 0.0001 |
|  |  |  |  | IVM 0 µM vs. PAL 25 µM | 0.3200 | -0.3133 to 0.9533 | 0.2333 |
|  |  |  | MCF-7/LCC9 | IVM 0 µM vs. IVM 3 µM | -0.0633 | -0.4420 to 0.3154 | 0.9299 |
|  |  |  |  | IVM 0 µM vs. IVM 6 µM | 0.2400 | -0.1387 to 0.6187 | 0.2343 |
|  |  |  |  | IVM 0 µM vs. IVM 9 µM | 0.4867 | 0.1080 to 0.8654 | 0.0152 |
|  |  |  |  | IVM 0 µM vs. PAL 25 µM | 0.1300 | -0.6095 to 0.8695 | 0.6510 |
| Figure 5D | Western Blot | Axin1 | MCF-7/LCC2 | IVM 0 µM vs. IVM 3 µM | -0.1633 | -0.7651 to 0.4384 | 0.4930 |
|  |  |  |  | IVM 0 µM vs. IVM 6 µM | -0.5200 | -0.8716 to -0.1684 | 0.0148 |
|  |  |  |  | IVM 0 µM vs. IVM 9 µM | -0.4300 | -0.7081 to -0.1519 | 0.0127 |
|  |  |  |  | IVM 0 µM vs. PAL 25 µM | -0.0933 | -0.8308 to 0.6441 | 0.7430 |
|  |  |  | MCF-7/LCC9 | IVM 0 µM vs. IVM 3 µM | -0.0400 | -0.5714 to 0.4914 | 0.8447 |
|  |  |  |  | IVM 0 µM vs. IVM 6 µM | -0.2967 | -0.5101 to -0.0832 | 0.0182 |
|  |  |  |  | IVM 0 µM vs. IVM 9 µM | -0.6067 | -0.7868 to -0.4265 | 0.0007 |
|  |  |  |  | IVM 0 µM vs. PAL 25 µM | 0.0567 | -0.4168 to 0.5301 | 0.7563 |

| **Figure** | | **Assay** | | **Experimental detail** | **Cell lines** | | | **Group comparisons** | **Mean Diff.** | | **95% CI of diff.** | | | **P value** |
| --- | --- | --- | --- | --- | --- | --- | --- | --- | --- | --- | --- | --- | --- | --- |
| Figure S1 (A) | | MTT | | Combined treatment:  at 4-OHT 0 µM | MCF-7/LCC2 | | | IVM 0 µM vs. IVM 4 µM | 47.47 | | 36.73 to 58.21 | | | <0.0001 |
|  |  |  |  |  |  |  |  | IVM 0 µM vs. IVM 7 µM | 65.58 | | 54.84 to 76.32 | | | <0.0001 |
|  |  |  |  |  |  |  |  | IVM 0 µM vs. IVM 9 µM | 75.54 | | 64.81 to 86.28 | | | <0.0001 |
|  |  |  |  | Combined treatment:  at 4-OHT 2.5 µM | MCF-7/LCC2 | | | IVM 0 µM vs. IVM 4 µM | 34.64 | | 23.91 to 45.38 | | | <0.0001 |
|  |  |  |  |  |  |  |  | IVM 0 µM vs. IVM 7 µM | 49.86 | | 39.12 to 60.60 | | | <0.0001 |
|  |  |  |  |  |  |  |  | IVM 0 µM vs. IVM 9 µM | 55.29 | | 44.55 to 66.02 | | | <0.0001 |
|  |  |  |  | Combined treatment:  at 4-OHT 5 µM | MCF-7/LCC2 | | | IVM 0 µM vs. IVM 4 µM | 45.24 | | 34.50 to 55.97 | | | <0.0001 |
|  |  |  |  |  |  |  |  | IVM 0 µM vs. IVM 7 µM | 50.99 | | 40.26 to 61.73 | | | <0.0001 |
|  |  |  |  |  |  |  |  | IVM 0 µM vs. IVM 9 µM | 57.58 | | 46.85 to 68.32 | | | <0.0001 |
|  |  |  |  | Combined treatment:  at 4-OHT 7.5 µM | MCF-7/LCC2 | | | IVM 0 µM vs. IVM 4 µM | 51.16 | | 40.42 to 61.90 | | | <0.0001 |
|  |  |  |  |  |  |  |  | IVM 0 µM vs. IVM 7 µM | 64.27 | | 53.53 to 75.00 | | | <0.0001 |
|  |  |  |  |  |  |  |  | IVM 0 µM vs. IVM 9 µM | 68.44 | | 57.70 to 79.18 | | | <0.0001 |
|  |  |  |  | Combined treatment:  at 4-OHT 10 µM | MCF-7/LCC2 | | | IVM 0 µM vs. IVM 4 µM | 15.54 | | 4.799 to 26.27 | | | 0.0030 |
|  |  |  |  |  |  |  |  | IVM 0 µM vs. IVM 7 µM | 30.53 | | 19.79 to 41.26 | | | <0.0001 |
|  |  |  |  |  |  |  |  | IVM 0 µM vs. IVM 9 µM | 30.91 | | 20.17 to 41.65 | | | <0.0001 |
| Figure S1 (B) | | MTT | | Combined treatment:  at 4-OHT 0 µM | MCF-7/LCC9 | | | IVM 0 µM vs. IVM 3 µM | 36.17 | | 27.61 to 44.73 | | | <0.0001 |
|  |  |  |  |  |  |  |  | IVM 0 µM vs. IVM 5 µM | 62.23 | | 53.67 to 70.79 | | | <0.0001 |
|  |  |  |  |  |  |  |  | IVM 0 µM vs. IVM 7 µM | 77.84 | | 69.28 to 86.40 | | | <0.0001 |
|  |  |  |  | Combined treatment:  at 4-OHT 2.5 µM | MCF-7/LCC9 | | | IVM 0 µM vs. IVM 3 µM | 32.33 | | 23.77 to 40.89 | | | <0.0001 |
|  |  |  |  |  |  |  |  | IVM 0 µM vs. IVM 5 µM | 46.46 | | 37.90 to 55.02 | | | <0.0001 |
|  |  |  |  |  |  |  |  | IVM 0 µM vs. IVM 7 µM | 52.84 | | 44.28 to 61.40 | | | <0.0001 |
|  |  |  |  | Combined treatment:  at 4-OHT 5 µM | MCF-7/LCC9 | | | IVM 0 µM vs. IVM 3 µM | 30.51 | | 21.95 to 39.07 | | | <0.0001 |
|  |  |  |  |  |  |  |  | IVM 0 µM vs. IVM 5 µM | 53.69 | | 45.13 to 62.25 | | | <0.0001 |
|  |  |  |  |  |  |  |  | IVM 0 µM vs. IVM 7 µM | 59.01 | | 50.45 to 67.57 | | | <0.0001 |
|  |  |  |  | Combined treatment:  at 4-OHT 7.5 µM | MCF-7/LCC9 | | | IVM 0 µM vs. IVM 3 µM | 33.33 | | 24.77 to 41.89 | | | <0.0001 |
|  |  |  |  |  |  |  |  | IVM 0 µM vs. IVM 5 µM | 55.58 | | 47.02 to 64.14 | | | <0.0001 |
|  |  |  |  |  |  |  |  | IVM 0 µM vs. IVM 7 µM | 59.16 | | 50.60 to 67.72 | | | <0.0001 |
|  |  |  |  | Combined treatment:  at 4-OHT 10 µM | MCF-7/LCC9 | | | IVM 0 µM vs. IVM 3 µM | 16.65 | | 8.091 to 25.21 | | | <0.0001 |
|  |  |  |  |  |  |  |  | IVM 0 µM vs. IVM 5 µM | 21.41 | | 12.85 to 29.97 | | | <0.0001 |
|  |  |  |  |  |  |  |  | IVM 0 µM vs. IVM 7 µM | 22.30 | | 13.74 to 30.86 | | | <0.0001 |
| **Figure** | **Assay** | | **Experimental detail** | | | **Cell lines** | **Group comparisons** | | | **Mean Diff.** | | **95% CI of diff.** | **P value** | |
| Figure S1 (C) | MTT | | Combined treatment:  at 4-OHT 0 µM | | | MCF-7 | IVM 0 µM vs. IVM 0.5 µM | | | 13.7 | | -1.181 to 28.59 | 0.0770 | |
|  |  |  |  |  |  |  | IVM 0 µM vs. IVM 2 µM | | | 2.023 | | -12.86 to 16.91 | 0.9755 | |
|  |  |  |  |  |  |  | IVM 0 µM vs. IVM 8 µM | | | 63 | | 48.11 to 77.88 | <0.0001 | |
|  |  |  | Combined treatment:  at 4-OHT 2.5 µM | | | MCF-7 | IVM 0 µM vs. IVM 0.5 µM | | | -3.23 | | -18.16 to 11.70 | 0.9152 | |
|  |  |  |  |  |  |  | IVM 0 µM vs. IVM 2 µM | | | -13.8 | | -30.49 to 2.885 | 0.1248 | |
|  |  |  |  |  |  |  | IVM 0 µM vs. IVM 8 µM | | | 32.81 | | 17.89 to 47.74 | <0.0001 | |
|  |  |  | Combined treatment:  at 4-OHT 5 µM | | | MCF-7 | IVM 0 µM vs. IVM 0.5 µM | | | 0.2867 | | -14.60 to 15.17 | >0.9999 | |
|  |  |  |  |  |  |  | IVM 0 µM vs. IVM 2 µM | | | 6.657 | | -8.228 to 21.54 | 0.5651 | |
|  |  |  |  |  |  |  | IVM 0 µM vs. IVM 8 µM | | | 49.03 | | 34.15 to 63.92 | <0.0001 | |
|  |  |  | Combined treatment:  at 4-OHT 7.5 µM | | | MCF-7 | IVM 0 µM vs. IVM 0.5 µM | | | 8.87 | | -6.014 to 23.75 | 0.3413 | |
|  |  |  |  |  |  |  | IVM 0 µM vs. IVM 2 µM | | | 12.08 | | -2.808 to 26.96 | 0.1340 | |
|  |  |  |  |  |  |  | IVM 0 µM vs. IVM 8 µM | | | 48.3 | | 33.41 to 63.18 | <0.0001 | |
|  |  |  | Combined treatment:  at 4-OHT 10 µM | | | MCF-7 | IVM 0 µM vs. IVM 0.5 µM | | | 12.8 | | -2.081 to 27.69 | 0.1052 | |
|  |  |  |  |  |  |  | IVM 0 µM vs. IVM 2 µM | | | 12.77 | | -2.114 to 27.65 | 0.1064 | |
|  |  |  |  |  |  |  | IVM 0 µM vs. IVM 8 µM | | | 28.39 | | 13.50 to 43.27 | 0.0001 | |

| **Figure** | **Assay** | **Experimental detail/ Target Protein** | **Cell lines** | **Group comparisons** | **Mean Diff.** | **95% CI of diff.** | **P value** |
| --- | --- | --- | --- | --- | --- | --- | --- |
| Figure S2 (B) | Western Blot | N-Cadherin | MCF-7/LCC2 | IVM 0 µM vs. IVM 3 µM | 0.0967 | -0.4796 to 0.6729 | 0.9294 |
|  |  |  |  | IVM 0 µM vs. IVM 6 µM | -0.0700 | -0.6463 to 0.5063 | 0.9703 |
|  |  |  |  | IVM 0 µM vs. IVM 9 µM | 0.1567 | -0.4196 to 0.7329 | 0.7783 |
|  |  |  |  | IVM 0 µM vs. PAL 25 µM | -0.1167 | -0.3301 to 0.09680 | 0.2038 |
|  |  |  | MCF-7/LCC9 | IVM 0 µM vs. IVM 3 µM | -0.2567 | -1.191 to 0.6781 | 0.7737 |
|  |  |  |  | IVM 0 µM vs. IVM 6 µM | -0.0267 | -0.9614 to 0.9081 | 0.9996 |
|  |  |  |  | IVM 0 µM vs. IVM 9 µM | -0.5000 | -1.435 to 0.4347 | 0.3443 |
|  |  |  |  | IVM 0 µM vs. PAL 25 µM | -0.1300 | -0.3939 to 0.1339 | 0.2432 |
| Figure S2 (C) | Western Blot | β-Catenin | MCF-7/LCC2 | IVM 0 µM vs. IVM 3 µM | -0.0233 | -0.6844 to 0.6378 | 0.9992 |
|  |  |  |  | IVM 0 µM vs. IVM 6 µM | 0.0733 | -0.5878 to 0.7344 | 0.9769 |
|  |  |  |  | IVM 0 µM vs. IVM 9 µM | 0.2833 | -0.3778 to 0.9444 | 0.5018 |
|  |  |  |  | IVM 0 µM vs. PAL 25 µM | -0.1100 | -0.3221 to 0.1021 | 0.2232 |
|  |  |  | MCF-7/LCC9 | IVM 0 µM vs. IVM 3 µM | 0.2533 | -0.3254 to 0.8321 | 0.4868 |
|  |  |  |  | IVM 0 µM vs. IVM 6 µM | 0.2167 | -0.3621 to 0.7954 | 0.5952 |
|  |  |  |  | IVM 0 µM vs. IVM 9 µM | 0.3700 | -0.2088 to 0.9488 | 0.2291 |
|  |  |  |  | IVM 0 µM vs. PAL 25 µM | -0.3900 | -0.5685 to -0.2115 | 0.0037 |
| Figure S2 (D) | Western Blot | Naked1 | MCF-7/LCC2 | IVM 0 µM vs. IVM 3 µM | -0.1333 | -0.3041 to 0.0374 | 0.1274 |
|  |  |  |  | IVM 0 µM vs. IVM 6 µM | 0.0233 | -0.1474 to 0.1941 | 0.9590 |
|  |  |  |  | IVM 0 µM vs. IVM 9 µM | 0.0367 | -0.1341 to 0.2074 | 0.8695 |
|  |  |  |  | IVM 0 µM vs. PAL 25 µM | -0.1800 | -0.5590 to 0.1990 | 0.2577 |
|  |  |  | MCF-7/LCC9 | IVM 0 µM vs. IVM 3 µM | -0.3267 | -0.9854 to 0.3321 | 0.3976 |
|  |  |  |  | IVM 0 µM vs. IVM 6 µM | -0.4467 | -1.1050 to 0.2121 | 0.1957 |
|  |  |  |  | IVM 0 µM vs. IVM 9 µM | -0.1833 | -0.8421 to 0.4754 | 0.7672 |
|  |  |  |  | IVM 0 µM vs. PAL 25 µM | 0.1033 | -0.01804 to 0.2247 | 0.0773 |

| **Figure** | **Assay** | **Experimental detail/ Target Protein** | **Cell lines** | **Group comparisons** | **Mean Diff.** | **95% CI of diff.** | **P value** |
| --- | --- | --- | --- | --- | --- | --- | --- |
| Figure S2 (E) | Western Blot | Naked2 | MCF-7/LCC2 | IVM 0 µM vs. IVM 3 µM | -0.2633 | -0.7125 to 0.1858 | 0.2828 |
|  |  |  |  | IVM 0 µM vs. IVM 6 µM | 0.1000 | -0.3492 to 0.5492 | 0.8579 |
|  |  |  |  | IVM 0 µM vs. IVM 9 µM | 0.0333 | -0.4158 to 0.4825 | 0.9927 |
|  |  |  |  | IVM 0 µM vs. PAL 25 µM | 0.4033 | -0.1566 to 0.9633 | 0.1161 |
|  |  |  | MCF-7/LCC9 | IVM 0 µM vs. IVM 3 µM | -0.1700 | -0.8902 to 0.5502 | 0.8374 |
|  |  |  |  | IVM 0 µM vs. IVM 6 µM | -0.1733 | -0.8935 to 0.5468 | 0.8300 |
|  |  |  |  | IVM 0 µM vs. IVM 9 µM | 0.1700 | -0.5502 to 0.8902 | 0.8374 |
|  |  |  |  | IVM 0 µM vs. PAL 25 µM | 0.3133 | -0.5150 to 1.1420 | 0.3529 |
| Figure S2 (F) | Western Blot | Dvl2 | MCF-7/LCC2 | IVM 0 µM vs. IVM 3 µM | -0.1767 | -1.150 to 0.7963 | 0.9135 |
|  |  |  |  | IVM 0 µM vs. IVM 6 µM | -0.3467 | -1.320 to 0.6263 | 0.6275 |
|  |  |  |  | IVM 0 µM vs. IVM 9 µM | -0.1900 | -1.163 to 0.7829 | 0.8963 |
|  |  |  |  | IVM 0 µM vs. PAL 25 µM | 0.5100 | -0.0883 to 1.1080 | 0.0771 |
|  |  |  | MCF-7/LCC9 | IVM 0 µM vs. IVM 3 µM | -0.1200 | -0.8278 to 0.5878 | 0.9274 |
|  |  |  |  | IVM 0 µM vs. IVM 6 µM | 0.0333 | -0.6745 to 0.7411 | 0.9981 |
|  |  |  |  | IVM 0 µM vs. IVM 9 µM | 0.5533 | -0.1545 to 1.2610 | 0.1269 |
|  |  |  |  | IVM 0 µM vs. PAL 25 µM | 0.0833 | -0.1893 to 0.3560 | 0.4439 |
| Figure S2 (G) | Western Blot | Dvl3 | MCF-7/LCC2 | IVM 0 µM vs. IVM 3 µM | 0.1167 | -0.2391 to 0.4725 | 0.6788 |
|  |  |  |  | IVM 0 µM vs. IVM 6 µM | 0.2733 | -0.0825 to 0.6291 | 0.1344 |
|  |  |  |  | IVM 0 µM vs. IVM 9 µM | 0.0267 | -0.3291 to 0.3825 | 0.9925 |
|  |  |  |  | IVM 0 µM vs. PAL 25 µM | 0.1367 | -0.1547 to 0.4280 | 0.2627 |
|  |  |  | MCF-7/LCC9 | IVM 0 µM vs. IVM 3 µM | -0.1233 | -0.4559 to 0.2092 | 0.6013 |
|  |  |  |  | IVM 0 µM vs. IVM 6 µM | -0.2733 | -0.6059 to 0.0592 | 0.1070 |
|  |  |  |  | IVM 0 µM vs. IVM 9 µM | -0.1667 | -0.4992 to 0.1659 | 0.3899 |
|  |  |  |  | IVM 0 µM vs. PAL 25 µM | 0.3833 | -0.2011 to 0.9678 | 0.1427 |

| **Figure** | **Assay** | **Experimental detail/ Target Protein** | **Cell lines** | **Group comparisons** | **Mean Diff.** | **95% CI of diff.** | **P value** |
| --- | --- | --- | --- | --- | --- | --- | --- |
| Figure S3 (B) | Western Blot | Snail | MCF-7 | IVM 0 µM vs. IVM 3 µM | 0.1900 | -0.06891 to 0.4489 | 0.1552 |
|  |  |  |  | IVM 0 µM vs. IVM 6 µM | 0.6900 | 0.4311 to 0.9489 | 0.0002 |
|  |  |  |  | IVM 0 µM vs. IVM 9 µM | 0.6100 | 0.3511 to 0.8689 | 0.0004 |
|  |  |  |  | IVM 0 µM vs. PAL 25 µM | -0.5633 | -0.7879 to -0.3387 | 0.0022 |
| Figure S4 (B) | Western Blot | LRP6 | MCF-7 | IVM 0 µM vs. IVM 3 µM | -0.0267 | -0.4240 to 0.3706 | 0.8612 |
|  |  |  |  | IVM 0 µM vs. IVM 6 µM | -0.2500 | -0.5416 to 0.04164 | 0.0760 |
|  |  |  |  | IVM 0 µM vs. IVM 9 µM | -0.5833 | -1.148 to -0.01856 | 0.0456 |
|  |  |  |  | IVM 0 µM vs. PAL 25 µM | 0.2867 | -0.7068 to 1.280 | 0.4679 |
| Figure S4 (C) | Western Blot | Axin1 | MCF-7 | IVM 0 µM vs. IVM 3 µM | -0.0333 | -0.1955 to 0.1288 | 0.5987 |
|  |  |  |  | IVM 0 µM vs. IVM 6 µM | -0.1433 | -0.8467 to 0.5600 | 0.6018 |
|  |  |  |  | IVM 0 µM vs. IVM 9 µM | -0.2633 | -0.5759 to 0.04928 | 0.0795 |
|  |  |  |  | IVM 0 µM vs. PAL 25 µM | -0.1200 | -0.5714 to 0.3314 | 0.5014 |
| Figure S5 (B) | Western Blot | N-Cadherin | MCF-7 | IVM 0 µM vs. IVM 3 µM | -0.6700 | -1.352 to 0.01208 | 0.0539 |
|  |  |  |  | IVM 0 µM vs. IVM 6 µM | -0.2900 | -0.9721 to 0.3921 | 0.5074 |
|  |  |  |  | IVM 0 µM vs. IVM 9 µM | -0.1200 | -0.8021 to 0.5621 | 0.9202 |
|  |  |  |  | IVM 0 µM vs. PAL 25 µM | 0.1833 | -0.04804 to 0.4147 | 0.0927 |
| Figure S5 (C) | Western Blot | β-Catenin | MCF-7 | IVM 0 µM vs. IVM 3 µM | -0.2867 | -1.095 to 0.5221 | 0.6308 |
|  |  |  |  | IVM 0 µM vs. IVM 6 µM | -0.0833 | -0.8921 to 0.7254 | 0.9813 |
|  |  |  |  | IVM 0 µM vs. IVM 9 µM | 0.1267 | -0.6821 to 0.9354 | 0.9410 |
|  |  |  |  | IVM 0 µM vs. PAL 25 µM | 0.1800 | -0.5014 to 0.8614 | 0.5040 |
| Figure S5 (D) | Western Blot | Naked1 | MCF-7 | IVM 0 µM vs. IVM 3 µM | -0.3200 | -1.254 to 0.6140 | 0.6522 |
|  |  |  |  | IVM 0 µM vs. IVM 6 µM | -0.3167 | -1.251 to 0.6173 | 0.6587 |
|  |  |  |  | IVM 0 µM vs. IVM 9 µM | -0.0733 | -1.007 to 0.8607 | 0.9914 |
|  |  |  |  | IVM 0 µM vs. PAL 25 µM | 0.1100 | -0.1419 to 0.3619 | 0.2921 |

| **Figure** | **Assay** | **Experimental detail/ Target Protein** | **Cell lines** | **Group comparisons** | **Mean Diff.** | **95% CI of diff.** | **P value** |
| --- | --- | --- | --- | --- | --- | --- | --- |
| Figure S5 (E) | Western Blot | Naked2 | MCF-7 | IVM 0 µM vs. IVM 3 µM | 0.0100 | -1.153 to 1.173 | >0.9999 |
|  |  |  |  | IVM 0 µM vs. IVM 6 µM | 0.1167 | -1.047 to 1.280 | 0.9827 |
|  |  |  |  | IVM 0 µM vs. IVM 9 µM | 0.2800 | -0.8835 to 1.443 | 0.8301 |
|  |  |  |  | IVM 0 µM vs. PAL 25 µM | 0.0467 | -0.8493 to 0.9427 | 0.8920 |
| Figure S5 (F) | Western Blot | Dvl2 | MCF-7 | IVM 0 µM vs. IVM 3 µM | -0.2933 | -1.038 to 0.4513 | 0.5607 |
|  |  |  |  | IVM 0 µM vs. IVM 6 µM | -0.1900 | -0.9346 to 0.5546 | 0.8066 |
|  |  |  |  | IVM 0 µM vs. IVM 9 µM | 0.1267 | -0.6180 to 0.8713 | 0.9267 |
|  |  |  |  | IVM 0 µM vs. PAL 25 µM | 0.4400 | -1.206 to 2.086 | 0.4992 |
| Figure S5 (G) | Western Blot | Dvl3 | MCF-7 | IVM 0 µM vs. IVM 3 µM | 0.1167 | -0.6368 to 0.8702 | 0.9427 |
|  |  |  |  | IVM 0 µM vs. IVM 6 µM | 0.1233 | -0.6302 to 0.8768 | 0.9337 |
|  |  |  |  | IVM 0 µM vs. IVM 9 µM | 0.0367 | -0.7168 to 0.7902 | 0.9979 |
|  |  |  |  | IVM 0 µM vs. PAL 25 µM | 0.4267 | -0.4428 to 1.296 | 0.2447 |
